# A Systematic Review of Associations between Amount of Meditation Practice and Outcomes in Interventions Using the Four Immeasurables Meditations

**DOI:** 10.3389/fpsyg.2017.00141

**Published:** 2017-02-06

**Authors:** Xianglong Zeng, Floria H. N. Chio, Tian P. S. Oei, Freedom Y. K. Leung, Xiangping Liu

**Affiliations:** ^1^School of Psychology, Bejing Normal UniversityBeijing, China; ^2^Department of Psychology, The Chinese University of Hong KongHong Kong, Hong Kong; ^3^School of Psychology, University of QueenslandBrisbane, QLD, Australia; ^4^Department of Psychology, James Cook University SingaporeSingapore, Singapore; ^5^Department of Psychology, Nanjing UniversityNanjing, China

**Keywords:** loving-kindness meditation, compassion, appreciative joy, active component, time of practice, dose response relationship, Buddhism

## Abstract

Interventions using the “Four Immeasurables Meditations” (FIM) are effective for various outcomes; however, whether increased meditation practice in these interventions leads to better results has not been well investigated. This systematic review included 22 FIM interventions that reported associations between the amount of meditation practice and its outcomes. Despite the heterogeneity in intervention components and outcome variables, there were generally few significant associations between amount of meditation practice and its outcomes. Specifically, only five studies reported that more than half of the calculated results were significant. In comparison with correlations between total amount of practice and overall outcomes, the short-term influence of meditation practice was evaluated in fewer studies; however, it had a better association with outcomes. More studies are required that address the underlying mechanisms that elucidate how meditation practice leads to outcome changes in daily life. In this study, two promising mechanisms with initial evidence were discussed. This review also summarized common methodological issues including a lack of experimental manipulation and inaccurate measuring of meditation practice.

## Introduction

Buddhism emphasizes the cultivation of four “sublime” or “noble” attitudes toward all beings: loving-kindness (friendliness), compassion (willing to cease suffering), appreciative joy (feeling happy for others), and equanimity (calm based on wisdom). These are known as the “four immeasurables” (Sujiva, 2007). These are cultivated by “Four Immeasurables Meditations” (FIM), where practitioners imagine certain targets (e.g., a person) and generate an “immeasurable” to this target. Each immeasurable is cultivated by one FIM subtype: loving-kindness meditation (LKM) generally wishes a person to be happy and to develop loving-kindness; compassion meditation (CM) wishes a suffering person to eliminate suffering, thus developing compassion; appreciative joy meditation (AJM) wishes a successful person not to lose success or to become more successful; and equanimity meditation (EM) develops a calm attitude toward the fate of the imagined target with wisdom in Buddhism (e.g., karma; Sujiva, 2007; Kraus and Sears, 2009; Hofmann et al., 2011; Zeng et al., 2015). Additionally, each FIM also seeks to reduce one negative attitude that is the opposite or “direct enemy” of each FI: LKM targets anger, CM targets hatred, AJM targets envy, and EM targets attachment (Sujiva, 2007; Kraus and Sears, 2009). The technical details of FIM vary across different Buddhist traditions. Most traditions silently repeat blessing phrases toward imagined targets (e.g., “may you be happy” in LKM). Some traditions also use imagination such as visualizing golden light radiating from oneself toward targets to generate a specific attitude (Sujiva, 2007; Zeng et al., 2015). Table 1 briefly summarized the practice and purpose of FIM's four subtypes per the Theravada tradition (Sujiva, 2007).

**Table 1:** **Mental activities and purposes of different four immeasurables meditations**.

**FIM**	**Imagined targets**	**Examples of blessing phrases**	**FI to be cultivated**	**Direct enemy to be reduced**
LKM	People with neutral emotions or peaceful smiling	“May he/she be free from enmity/danger”/”May he/she take care of himself/herself happily” (Sujiva, 2007, p. 21–22)	Loving-kindness	Anger
CM	People in success or happiness	“May he be free from mental suffering”/”May he be free from physical suffering” (Sujiva, 2007, p. 67)	Compassion	Hatred
AJM	People in suffering or sadness	“May he not cease from having whatever material gains acquired.”/”May he continue to have whatever spiritual happiness attained and may he gain even more” (Sujiva, 2007, p. 71)	Appreciative Joy	Envy
EM	People in good or bad experience	“He is the owner of his own kamma” (Sujiva, 2007, p. 76)	Equanimity	Attachment

Empirical studies on the effects of FIM have increased sharply in recent years, and a series of studies evaluated the effects of interventions based on FIM on various outcomes (Galante et al., 2014). Recent systematic reviews summarized these interventions' effects on positive emotions (Zeng et al., 2015) and in clinical samples (Shonin et al., 2015). Additionally, some studies compared long-term FIM practitioners with naïve individuals (e.g., Lutz et al., 2004) or evaluated the effect of one-shot FIM practice in a laboratory setting (e.g., Hutcherson et al., 2008). These studies supported FIM's effectiveness using different approaches.

Meditation practices, regardless of type, consume much time, and energy. Experienced meditators in previous studies often had more than 10,000 h of meditation practice (e.g., Lutz et al., 2004). Many meditation-based interventions also require practice at home and encourage continuous practice after the intervention ends. Most Buddhist traditions believe substantial change of mind requires long-term meditation practice (e.g., Sujiva, 2007), and neuroscientists used neuroplasticity to explain the impact of repeated meditation practice (Davidson and Lutz, 2008). Although both traditional Buddhist teaching and innovative neuroscience supported the notion that repeated meditation practice is impactful, detailed questions such as, “How long is meditation practice necessary” have only been investigated in recent years (Carmody and Baer, 2009); therefore, they are not well answered yet. Furthermore, it is notable that many meditation-based interventions consist not only of meditation practice, but also of other components such as disclosure, where leaders teach both meditation skills and relevant philosophical ideas or Buddhist knowledge, and discussion, where participants share experiences and asked questions. A recent study found that a loving-kindness discussion group without any meditation can also change one's attitude toward one's self (Kang et al., 2015). Such findings also raise questions about how much of the overall outcomes should be attributed to meditation practice. Overall, an investigation of the relationship between amount of meditation practice and outcomes is very important as it benefits the best practice and improves the theoretical understanding on interventions' effects.

In their meta-analysis on FIM, Zeng et al. (2015) reported that the intervention length and time spent on meditation practice did not significantly predict the effect on positive emotions; however, they also noted that the meta-regression they used had low statistical power. They also mentioned a relationship between meditation practice and effects at the level of individual studies; however, their introduction to these individual studies was very brief, and different subtypes of FIM were not separated. Furthermore, Zeng et al. (2015) had limited outcome variables related to positive emotions, and there have been no other reviews discussing the association between the length or strength of meditation practices and outcome effects. Consequently, this article will provide a systematic and narrative review of empirical studies on FIM that reported a relationship between outcome variables and the amount of meditation practice (e.g., hours spent on meditation, number of times meditating). In addition to providing a comprehensive summary of current findings, a more important function of the detailed narrative review is to illustrate perspectives or assumptions used in previous research and their methodological shortcomings, which will benefit future research in this area.

## Methods

### Literature search

The databases search, which was completed on Feb 23, 2016, included MedlinePlus, ISI Core Journals, PsycInfo, Embase, CINAHL Plus, AMED, and Cochrane Central Register of Controlled Trials. The search set in the title, abstract, and keywords sections was “immeasurable OR immeasurables OR kindness OR compassion OR compassionate OR [(Appreciating OR Appreciative OR Sympathetic OR Empathic) AND Joy] OR equanimity OR metta OR mudita OR karuna OR upekkha” combined with “Meditat^*^ OR Buddhis^*^,” which were adjusted for different databases.

### Selection of studies

The inclusion criteria were (a) articles published in a peer-reviewed journal in English, (b) empirical studies addressing interventions wherein at least 50% of major practices were FIM, and (c) studies that reported a relationship between the amount of meditation practice and other variables.

### Data extraction and synthesis

The first two authors reviewed the titles and abstracts of unduplicated records to identify potential relevant articles for full text review; then, they extracted information about the amount of meditation practice from empirical studies on FIM. The reference lists of identified empirical studies were checked for missing articles. We attempted to contact the authors for unexplained selective reports; however, some researchers had not provided contact information or did not reply.

## Results

### Literature search result

Non-duplicated records (*N* = 1509) were collected and 135 articles were obtained for full text review. Among them, 77 articles were identified as empirical studies on FIM, and one more article (Rana, 2015) was added from references. Specifically, 13 empirical studies on FIM were identified in addition to those located in a June 2015 systematic literature search (Zeng et al., 2015). The supplemental materials list all articles for full-text viewing and illustrate the reasons for exclusion.

Among the 78 articles on FIM, 51 articles were concerned with interventions lasting 1 day or longer, and others included 14 cross-sectional studies among long-term FIM meditators and 14 one-shot practice sessions of FIM in laboratory settings. Among these FIM interventions, 20 articles reported a relationship between amount of meditation practice and outcomes, which will be reviewed below. Another 13 studies noted that they checked or recorded the practice time; however, they did not explore the relationship between practice time and its effects. The remaining studies on interventions did not mention practice time. The supplemental material lists the information for each article identified by the systematic literature search.

After the systematic literature search, another two articles (Leppma and Young, 2016; Weibel et al., 2016) that also reported a relationship between meditation practice amount and outcomes were published and discovered by this study's authors. Although these articles were not identified in the systematic search, this review includes them to provide the most current information. Therefore, in total, 22 articles will be reviewed below. Because most interventions focused on LKM or CM, the review below grouped interventions into LKM, CM, and others. Considering the purpose of this article, the narrative review below highlights meditation practice and its association with outcomes. Tables 2–**4** summarize details of additional information including intervention structure, demographic information, and detailed between-group differences.

**Table 2:** **Studies on interventions with loving-kindness meditation**.

**Study**	**Design (A)**	**Intervention**	**Participants**	**Results on group level (B)**	**Amount of Meditation practice**	**Effect of meditation practice (C)**
Carson et al., 2005	RCT: LKM vs. TAU; Pre-Post-FU	LKM: 8 weekly 90-min sessions, 10–30 min of practice at home every day	Patients with low back pain, *N* = 43 (Mean age = 51.1; 61% female; 18 in LKM, 25 in TAU). Unknown meditation experience	**Positive Effects:** (1) Pain (multiple scales), anxiety, psychological distress at pre-post (2) Variables above plus phobia and hostility at pre-follow up (3) Daily anger and daily tension throughout period of intervention **Non-significant Effects:** (1) Anger (multiple scales, trend significant) (2) Daily pain	Min spent on LKM practice every day: average 20.8 ± 6.3 min per day	**Significant Covariance:** Practice time was related to lower pain on the practice day and lower anger the next day **Non-significant Covariance:** Practice time was not related to daily tension Not all outcomes were calculated
Cohn and Fredrickson, 2010	15 month longitudinal results of Fredrickson et al., 2008	Same as Fredrickson et al., 2008	Remained *N* = 95 (Mean age = 42.2; *SD* = 9.7; 61.1% female, 45 from LKM, 50 from control), 33 recoded as continuers who continued practice with multiple meditations	**Non-significant Effects:** (1) Difference in positive emotions between continuers and non-continuers did not change from last assessment (2) No change in resources (mindfulness, psychological well-being and social well-being, hope, ego-resilience, savoring, social support, self-other overlap, symptoms of illness, life satisfaction) from last assessment	Type and frequency of meditation after interventions, daily amount of meditation for 1 week: 33 recoded as continuers and 37% continuers practiced LKM. Daily minutes of practice for continuers were 5.9 for “occasionally,” 28.4 for “frequent” and 35.2 for “daily”	**Significant Covariance:** (1) Positive emotions correlated positively with number of minutes spent meditating per day (2) Positive emotions before intervention and early reactivity could predict continuers
Fredrickson et al., 2008	RCT: LKM vs. Waitlist Control, Pre-Post	LKM: six 60-min group sessions held over 7 weeks. Participants were asked to practice LKM at home at least 5 days per week. Control group received same intervention later, and the data were used	Working adults, *N* = 139 (age = 41; 59.8% female in intent to treatment (ITT) analysis; 60.8% female in per protocol analysis; 65.5% in completer analysis; 67 in LKM, 72 in control). Four participants had a meditation practice	**Positive Effects:** (1) Positive emotions; resources (i.e., mindfulness, pathways thinking, savoring the future, environmental mastery, self-acceptance, purpose in life, social support received, positive relations with others, and illness symptoms), life satisfaction, depressive symptoms **Non-significant Effects:** (1) Negative emotion, compassion (as a positive emotion that was specifically noted)	Minutes spent on “meditation, prayer, or solo spiritual activity” every day: average 80 min per week (higher in LKM group than control group), dropped to 60 min per week after interventions	**Significant Covariance:** Amount of practice was related to (1) baseline positive emotions (2) positive emotions for all time points except week 4, with stronger effect during later weeks of intervention (3) increased positive emotions during social interactions (4) higher life satisfaction, higher resources (mindfulness, pathways thinking, savoring the future, environmental mastery, self-acceptance, purpose in life, social support received, positive relations with others, and illness symptoms), and lower depressive symptoms (mediated by positive emotions, not direct prediction) **Non-significant Covariance:** Amount of practice was not related to negative emotions
May et al., 2011	Non-RCT: LKM group vs. non-meditation control group, Pre-Post	LKM: LKM group practiced LKM for at least 15 min per day, 4 days per week, for 8 weeks without weekly course	College students LKM: *N* = 13 (Mean age = 22.08, 76.9% female) Control: *N* = 14 (mean age = 23.21; 78.6% female). Unknown meditation experience	**Positive Effects:** (1) LKM group showed significant improvement in observing and describing dimensions of Mindfulness **Non-significant Effects:** (1) **[Beh]** No group difference in attentional blinks at post-interventions (2) **[Bio]** No group difference in ECG at post-interventions (3) No pre-post difference in positive and negative affect in LKM group	Sum of meditation time in minutes: 485.15 ± 71.31	**Non-significant Covariance:** Meditation time did not significantly correlate with changes in observing and describing Not all outcomes were calculated
Leppma and Young, 2016	Non-RCT: LKM group vs. interpersonal skill training, Pre-Post	LKM: 6 weekly 60 min group sessions. Required 10–20 min 3 or 4 times per week. Interpersonal skill training: 6 weekly course on interpersonal skill	Master level counseling students. *N* = 107 (mean age = 27.5, *SD* = 8.2 years; 88% females; “the treatment group was less than twice the size of the control group,” p. 301) 51 participants had tried meditation previously	**Positive Effects:** (1) Significant interaction on perspective taking and fantasy, LKM group showed significant increase in these variables (2) LKM group showed significant increase in empathic concern **Non-significant Effects:** (1) No significant interaction or with-in group change in personal distress	Not Available	**Significant Covariance:** More meditation time is associated with higher perspective taking **Non-significant Covariance:** Meditation time did not significantly correlate with other dimensions of empathy
Weibel et al., 2016	RCT: LKM vs. Waitlist Control, Pre-Post-8-week FU	LKM: 4 90-min group sessions. Participants were encouraged to practice LKM at home.	University student. *N* = 71 (Mean age = 19.1, *SD* = 1.17 years old; 77% females; 38 in LKM, 33 in Control) Unknown meditation experience	**Positive Effects:** (1) LKM group showed higher self-compassion and compassionate love than control group (2) LKM group showed significant decrease in anxiety	Time of meditation practice over prior week in minutes: 29 ± 34 at post measurement, 15 ± 29 at follow up	**Non-significant Covariance**: Meditation time did not significantly predict outcomes

***(A)** “LKM” refers to loving-kindness meditation. “TAU” refers to treatment as usual. “Pre,” “Post,” and “FU” refer to pre-treatment measurement, post-treatment measurement, and follow up measurement, respectively. **(B)** This column sums all the outcomes measured in the studies. Outcomes with significant results at any time point will no longer be listed as non-significant results at other time points; the results of sub-concepts are grouped (e.g., sensory pain and affective pain as pain) and are considered significant if parts of those results are significant. **[Beh]** and **[Bio]** refer to using a behavioral task with dependent variables and biological or physiological indicators, respectively. Other variables are self-reported daily variables. **(C)** This column only lists the effects of amount of practice that were reported in the articles; that is, those variables listed in group-level results (fourth column), but omitted here, are due to selective reporting*.

### Interventions based on LKM

In one of the earliest empirical studies on LKM, Carson et al. (2005) compared a LKM intervention with a treatment-as-usual control group in a randomized control trial (RCT) among patients with lower back pain. The 8-week intervention required 10–30 min of practice at home every day. While the control group did not exhibit any significant change, the LKM group reported significant reductions in pain, global psychological distress, anxiety, hostility, and phobias after the intervention. Before and after the LKM practice each day, the participants reported their pain, anger, and tension on a 0–100 single-item scale, and daily anger and tension were reduced during the intervention. The time spent on LKM practice every day was recorded and the mean practice time was 20.8 ± 6.3 min per day. The authors only evaluated the relationship between the time of daily meditation practice and daily outcomes with multilevel modeling, and the results showed that practice time of LKM on a given day significantly predicted decreased daily pain on that day and decreased daily anger the next day, but not daily tension.

Fredrickson et al. (2008) compared a LKM intervention group with a wait-list control group in an RCT among healthy adults. The intervention lasted 7 weeks and required 15–22 min of at-home practice at least 5 days per week. This study measured time spent on meditation practice, 19 specific emotions each day, four groups of resources (i.e., cognitive, psychological, physical, and social resources), and ultimate outcomes (i.e., life satisfaction and depression symptoms) before and after interventions. The results showed that LKM increased daily positive emotions, which in turn increased resources, and finally resulted in improved ultimate outcomes; however, no significant effect on negative emotions was found. The average meditation practice time was 80 min per week, and amount of meditation practice predicted daily positive emotions, but not daily negative emotions. Furthermore, the effects of practice time on increasing positive emotions were stronger in later weeks than in earlier weeks, which suggests that effects of LKM practice improved with time as practitioners performed LKM with increasing skill. It is also notable that the intention-to-treat analysis found that the amount of practice had no significant correlation with positive emotions; therefore, the findings above were restricted to individuals who invested adequate effort and the generalizability of the conclusions was limited. Although there was no direct effect, meditation practice time had effects on resources (see Table 2) and depressive symptoms via change in positive emotions. Additionally, based on the day-reconstruction method for certain morning-after interventions, Fredrickson et al. (2008) also found that both LKM practice time during the intervention and practice of LKM on the analyzed day predicted a higher frequency of positive emotions and that LKM practice time during the intervention also facilitated positive emotions gained from social interaction on the analyzed morning. A longitudinal report on this study (Cohn and Fredrickson, 2010) found that the improvements that were affected by the interventions remained 15 months later. However, whether meditation (not only LKM) continued or ceased did not further influence the results; in contrast, continuation, or cessation could be predicted by positive emotions before the interventions and earlier reactivity (the increase of positive emotions in the early part of the intervention). However, the participants' daily records for 1 week showed that the amount of daily meditation was still correlated with positive emotions.

May et al. (2011; experiment 1) evaluated effects of 8 weeks of LKM practice among university students. The participants were required to practice LKM at least 15 min per day for 4 days per week: the average sum of real meditation practice was 485.15 ± 71.31 min. The practitioners exhibited significant changes on the “observe” and “describe” dimensions of the mindfulness scale; however, these changes were not correlated with amount of meditation practice. Additionally, their daily positive or negative emotions were not changed after intervention, and their behavioral and ECG responses during an attentional blink task did not differ from those of the control group, who were further recruited without any training. However, the effects of amount of meditation practice on these outcomes were not reported.

Leppma and Young (2016) compared a LKM intervention with matched interpersonal skill training among Masters counseling students with a quasi-experimental design. The 6-week LKM intervention required 10–20 min of at home practice three or four times per week (obtained through personal communication with Leppma), but the actual length of practice was not specified. The primary outcome variable was empathy with multiple dimensions. The results showed a significant group × time interaction on perspective taking and fantasy, driven by the increase in these two dimensions in the LKM group. No group × time interaction was found in empathic concern and personal distress; however, the LKM group showed a significant increase in empathic concern. More meditation time was associated with higher perspective taking, but not other dimensions of empathy. Additional measurements that were not included in the primary research questions of this article were omitted here.

Weibel et al. (2016) compared a LKM intervention group with a wait-list control group in an RCT among university students. The LKM intervention consisted of 4 weekly 90-min group sessions. The participants were encouraged to practice at home and reported 29 ± 34 min of meditation practice over prior week at post-treatment and 15 ± 29 min at follow-up. After controlling for pre-treatment scores, the LKM group had significantly higher self-compassion and compassionate love than did the control group at post-treatment (but not follow-up). Anxiety showed significant difference between the two groups; however, it significantly decreased within the LKM group. The meditation practice time in the LKM group did not predict any outcome variable.

### Intervention based on CM

Six interventions adopted cognitive-based compassion training (CBCT; Pace et al., 2009), and the structure and length of the course varied across studies (see Table 2). The first intervention (Pace et al., 2009), compared CBCT with a matched health discussion group in an RCT among university students. This 6-week intervention required daily practice at home; however, the length of practice was not specified. After the interventions, the participants took a Trier Social Stress Test (TSST) where participants gave a presentation in front of experts. The subjective distress and biological change [Interleukin 6 (IL-6) and cortisol] were recorded; however, no significant group differences were found. The research team recorded the number of meditation sessions (including at-home sessions that exceeded 10 min and the total number of in-class meditation sessions); the weekly averages were 2.81 ± 1.65 sessions with 20.08 ± 4.54 min per at-home session. The analysis showed that the number of meditation sessions in the CBCT group was negatively associated with TSST-induced subjective distress and IL-6 increase, but not with cortisol response. Furthermore, participants with meditation practice times above the median showed lower TSST-induced IL-6 than participants below the median, and high-practice time meditators demonstrated reduced subjective distress before the stress induction than participants with low practice time and those in the control group.

The lack of baseline measurement in the first study (Pace et al., 2009) led to a second trial among university students. This time, no control group was used (Pace et al., 2010). The researchers showed that subjective distress, IL-6 increase, and cortisol response in TSST before the intervention did not predict the amount of meditation practice during the intervention; however, the sum of meditation practice was not reported. The amount of practice was positively correlated with age; however, the relationship between outcome and practice time was not changed after age was controlled for.

Desbordes et al. (2012) compared CBCT with matched mindful attention training and a health discussion control group in RCT among healthy adults. The 8-week CBCT required an average of 20 min of daily practice at home. The sum of meditation practice at home was 454 ± 205 min. No significant group × time interaction on self-reported anxiety and depression was found; however, the CBCT group showed a significant reduction in depression scores. As for brain activation in areas of the amygdala in response to pictures with different emotional valences in a non-meditative state measured by a functional magnetic resonance imaging (fMRI) scanner, the CBCT group showed no significant results for positive or neutral pictures, and the authors noted inconsistent results for negative pictures: activation in the right amygdala increased among those subjects with the most practice hours and decreased slightly among those with fewer practice hours (significance level unknown), and an increase in activation was significantly associated with a reduced depression score. However, no significant correlations between meditation practice and any outcome were confirmed in the CBCT group.

Mascaro et al. (2012) compared CBCT with a health-discussion control group in an RCT among community adults. The 8-week CBCT required 20 min of at-home practice each day. The self-reported practice of meditation was 315.9 ± 228.9 min in total. The CBCT group showed significantly higher accuracy in an empathy task (“Reading the Mind in the Eyes Test” (RMET) than the control group at post-intervention measurement, and no significant within-group difference in brain activation (although an interaction driven by the control group existed). However, no associations between amount of meditation practice and change of behavioral or brain activity was observed. In another report based on the same group of participants (Mascaro et al., 2013), the researchers reported results for the experienced empathy for pain (EFP) paradigm; however, no group × time interaction effect on brain activation or subjective ratings were found. The researchers found that the participants' pre-existing brain functioning could predict subsequent practice of mindfulness and compassion meditation in CBCT. Specifically, activation of the left amygdala during a “self-pain task” (i.e., participants received pain) was significantly correlated with practice time for mindfulness meditation, but not that for compassion meditation. Moreover, activation of the left and right anterior insula during the “other pain task” (i.e., participants viewed other people receiving pain in a video) was positively and significantly correlated with practice time during the compassion-specific portion, but not with mindfulness practice. No other correlations were found between the amount of practice and self-reported outcomes or demographic variables (sex, age, trait of empathy).

Another research program (Pace et al., 2013; Reddy et al., 2013) compared CBCT with a wait-list control group in an RCT among adolescents in the foster care system. The 6-week modified CBCT required 30 min of at-home practice every day. The weekly average times of meditation practice was 17.09 ± 20.69 times over the course, which increased from the first 3 weeks (7.8 ± 8.33 times) to the last three sessions (11.59 ± 16.37 times). Researchers measured several self-reported or parent-reported scales (see Table 2) and Salivary C-reactive protein before and after intervention; however, no between-group differences were found. The amounts of practice across interventions were correlated with reduced Salivary C-reactive protein, and the amounts of practice in the last 3 weeks were associated with an increase in hope and a trend-level decrease in the trait of anxiety. No other correlations were found for either the total practice amounts or those over the final 3 weeks. Notably, the items for emotion change before and after meditation every day were also used; however, they were not reported.

Dodds et al. (2015) compared CBCT with a wait-list control group in an RCT among a group of female breast cancer survivors. The 8-week CBCT required 30 min (on average) of at-home practice 3 times per week. The recorded sum of meditation practice was 738.5 ± 330.3 min across 8 weeks. The research group measured a series of self-reported scales and saliva cortisol as a biological indicator, and the group level changes (i.e., changes from pre- to post-intervention and at follow-up) are summarized in Table 3. Interestingly, the total meditation practice time was correlated with improvements in three outcomes (i.e., severity due to fear of cancer recurrence, psychological distress due to fear of cancer recurrence, and vitality) at the 4-week follow-up measurement, and none of these outcomes showed a significant difference at the group level. Notably, the authors noted that 25% of the participants in the CBCT group did not return their practice logs and that the average amount of practice was higher than in the previous CBCT intervention, which might have resulted in a ceiling effect.

**Table 3:** **Studies on interventions with compassion meditation**.

**Study**	**Design (A)**	**Intervention**	**Participants**	**Results on group level (B)**	**Amount of meditation practice**	**Effect of meditation practice (C)**
Desbordes et al., 2012	RCT: CBCT (Cognitive Based Compassion Training), Mindful attention training (MAT), and health discussion control group Pre-Post	CBCT & MAT: 2-h class per week for 8 weeks, with an average of 20 min a day outside the class for CBCT and MAT	Healthy adults, CBCT: *N* = 12 (Mean age = 32.0; *SD* = 5.4 years; 75% female); MAT: *N* = 12 (Mean age = 34.3; *SD* = 9.6; 66.67% female); Control: *N* = 12 (Mean age = 36; *SD* = 7.6; 41.67% female), no meditation experience	**Positive Effects:** (1) Decreased depression score (2) **[Neu]** Increase in activation in right amygdala for negative picture was correlated with reduced depression score **Non-significant Effects:** (1) **[Neu]** Activation in right amygdala had only trend decrease for positive picture, trend increase for negative images and no change for neutral picture	Sum of minutes of meditation practice at home, average 454 ± 205 min	**Significant Covariance:** Participants with most practice hour increased, while those with less practice decrease, in pre-post change in amygdala response to negative image (significance level unknown) **Non-significant Covariance:** Amount of practice was not correlated with any outcomes
Dodds et al., 2015	RCT: CBCT vs.wait-list control group. Pre-Post-4-week FU	CBCT: 8 weekly 2-h courses plus booster at 4 weeks after intervention. At-home practice three times per week, average length 30 min	Female breast cancer survivors CBCT: *N* = 12 (Mean age = 54.7; *SD* = 12.1 Control: *N* = 16 (Mean age = 55.8; *SD* = 9.7). 2 in CBCT reported often practicing meditation	**Positive Effects:** (1) Improvements in depression, functional impairment (in fear of cancer recurrence), avoidance (in traumatic stress), vitality and mindfulness at post intervention (2) Less perceived stress, higher mindfulness at follow up **Negative Effects:** Worse physical well-being at follow-up **Non-significant Effects:** (1) loneliness; gratitude and mental well-being; (2) **[Bio]** Saliva cortisol	Sum of meditation practice in minutes: average 738.5 ± 330.3 min across 8 weeks	**Significant Covariance:** Amount of practice was inversely correlated with severity and psychological distress (due to fear of cancer recurrence) at 4-week follow-up; it was also correlated with vitality at 4-week follow up **Non-significant Covariance:** Amount of practice was not correlated with all other outcomes at different time points
Hutcherson et al., 2015	RCT: CCT (Compassion Cultivation Training) vs. wait-list control group, Pre-Post	CCT: 8 2-h weekly classes, with requirement of 15 min of at-home practice every day	Community adults CCT: *N* = 50 (mean age = 41.98, *SD* = 11.48; 65% female) Control: *N* = 30 (mean age = 44.68, *SD* = 13.05; 80% females). Unknown meditation experience	**Positive Effects:** (1) Improvements in self-compassion and fear of compassion (for others, from others, for self) compared with control group	Average minutes of weekly formal meditation: 101.11 ± 57.00	**Significant Covariance:** Amount of formal meditation practice was associated with lower fear of compassion for others **Non-significant Covariance:** Amount of formal meditation practice was not associated with fear of compassion from others, fear of compassion for self and self-compassion
Jazaieri et al., 2014	Same as Jazaieri et al. (2013)	Same as Jazaieri et al. (2013)	Same as Jazaieri et al. (2013)	**Positive Effects:** (1) Mindfulness, decentering, worry, expressive suppression (frequency and self-efficacy) had group x time interaction **Non-significant Effects:** (1) Perceived stress, happiness, cognitive reappraisal (both frequency and self-efficacy) had no interaction	Same as Jazaieri et al., 2013	**Significant Covariance:** Amount of practice was associated with less worry and lower frequency of expressive suppression **Non-significant Covariance:** Amount of practice was not correlated with change in mindfulness, decentering, self-efficacy of expressive suppression, perceived stress, happiness and cognitive reappraisal
Jazaieri et al., 2015	Same as Jazaieri et al. (2013)	Same as Jazaieri et al. (2013)	CCT group in Jazaieri et al. (2013)	**Positive Effects:** (1) General and neutral mind-wandering, caring behavior for oneself **Non-significant Effects:** (1) Positive and negative mind-wandering, caring behavior for others	Whether meditation had been practiced that day, increase from 14.8% to 49.4% through interventions	**Significant Covariance:** Practice was associated with more positive and less negative mind-wandering, which in turn led to increase in caring behaviors for oneself and others **Non-significant Covariance:** Practice was not associated with neutral and general mind-wandering. No gender difference in amount of practice
Klimecki et al., 2012	Same as Leiberg et al. (2011)	Same as Leiberg et al. (2011)	Healthy female volunteers. *N* = 94 (Mean age = 24.3; *SD* = 4.17; 31 in CM, 33 in Memory, 30 additional participants not used). No meditation experience	**Positive Effects:** (1) [Beh] CM group but not memory group showed increased positive affect during high-emotion videos and empathy during low-emotion videos (2) **[Beh]** Correlation between empathy and negative affect was reduced in CM group but not in memory group (3) **[Beh/Neu]** CM group but not memory group showed stronger activation on mOFC, VTA/ SN, putamen, and pallidum (all right lateralized) in HE video but not LE video **Non-significant Effects:** (1) **[Beh]** Negative affect during video viewing	Hours of practice outside the training day: average 5.7	**Non-significant Covariance:** Amount of practice was not correlated with any dependent variables
Klimecki et al., 2013	Non-RCT: affect group vs. memory control group	Affect Group: Empathy training and a subsequent compassion training (both are 6-h 1-day courses with practice at home encouraged) Memory control group: Two matched training on memory skill.	Healthy female volunteers, Affect group: *N* = 25 (Mean age = 25.88, *SD* = 4.32) Memory group: *N* = 28 (Mean age: 22.89, *SD* = 4.02). No meditation experience	**Positive Effects:** (1) **[Beh]** Affect group showed higher empathy in the low-emotion videos than memory group (2) **[Beh]** Empathy training increased negative affect and empathy; subsequent compassion training increased positive affect and reduced increase in negative affect during video viewing (3) **[Beh/Neu]** Two stages in affective group involved different brain activation: empathy training increased activation in anterior insula and anteria midcingulate cortex, while compassion training increased activation in mOFC, pACC, IFG and ventral striatum	Duration of daily practice: 98.6 ± 60.91 min in empathy training, 65.72 ± 53.35 min in compassion training	**Non-significant Covariance**: Amount of practice was not correlated with any dependent variables
Leiberg et al., 2011	Non-RCT: CM training vs. memory training	CM training: 1-day 6-h compassion training with at least 2 h of at-home practice Memory training: matched training on memory skill	Healthy female volunteers, CM: *N* = 27 (Mean age = 24.74; *SD* = 4.22); Memory training: *N* = 32 (Mean age = 22.66; *SD* = 3.86). No meditation experience	**Positive Effects:** (1) CM group showed increase in compassionate love and decrease in negative moods (2) [Beh] CM group showed increase in helping in Zurich Prosocial Game (ZPG) **Non-significant Results:** (1) Both groups showed increased positive mood (2) [Beh] Neither group gave more money in dictator game	Hours of practice outside the training day: not available	**Significant Covariance:** Amount of at home practice was correlated with helping in no-reciprocity trials but not in other trials Not all outcomes were calculated
Mascaro et al., 2012	RCT: CBCT vs. health discussion control group. Pre-Post	CBCT: 2-h class per week for eight weeks. 20 min at-home practice each day Health discussion group: matched courses without any at-home practice	Community adults, CBCT: *N* = 13 (Mean age = 29.4; *SD* = 4.43) Control: *N* = 8 (Mean age = 35.9; *SD* = 8.06). Unknown meditation experience	**Positive Effects:** (1) **[Beh]** CBCT group had higher empathic accuracy than control group at post-training (2) **[Neu]** Significant interaction of time and group on activation in left inferior frontal gyrus and dorsomedial prefrontal cortex **Non-significant Effects:** (1) **[Neu]** Brain activation within CBCT group	Self-reported practice of meditation in minutes: 315.9 ± 228.9 total.	**Non-significant Covariance:** Amount of practice was not correlated with changes in empathic accuracy or brain activation
Mascaro et al., 2013	Same as Mascaro et al. (2012)	Same as Mascaro et al. (2012)	Same as Mascaro et al. (2012)	**Non-significant Effects:** (1) [Beh/Neu] No interaction effect on brain activation (2) [Beh] No interaction on subjective feeling of pain	Sum of daily practice of mindfulness and compassion meditations: average 53.1 ± 51.5 and 212.3 ± 190.3 min, respectively, not correlated with each other	**Significant Covariance:** (1) Amount of mindfulness practice was correlated with activity in the left amygdala during self-pain task (2) Amount of compassion practice was correlated with activity in the left and right anterior insula during other pain task **Non-significant Covariance:** Gender, age, trait of empathy or subjective ratings in task
Pace et al., 2009	RCT: CBCT vs. health discussion group. Post only	CBCT: participants attended 50-min class twice per week for 6 weeks Health discussion groups: once per week for 12 weeks during first-semester study groups, twice per week for six weeks in second-semester groups	University students, CBCT: *N* = 33 (Mean age = 18.48; *SD* = 0.62; 51.51% female); Control: *N* = 28 (Mean age = 18.54; *SD* = 0.69; 53.57% female). Unknown meditation experience	**Non-significant Effects:** (1) **[Beh/Bio]** No significant difference on IL-6, cortisol during TSST (2) **[Beh]** No significant difference on subjective distress during TSST	Average number of meditation sessions per week (including at-home sessions that exceeded 10 min and total number of in-class meditation sessions). 2.81 ± 1.65 sessions with 20.08 ± 4.54 min per at-home session. Control group attended significantly more classes than meditation group	**Significant Covariance:** (1) Practice time was correlated with decreased TSST-induced IL-6 and subjective distress; (while class attendance in control group had no significant correlations) (2) High-practice time meditators demonstrated reduced subjective distress before stress induction than participants with low practice time and those in control group (3) Participants with meditation practice times above median showed lower TSST-induced IL-6 and subjective distress scores than participants below median **Non-significant Covariance**: (1) Not correlated with maximal post-TSST cortisol (2) Not correlated with any demographic or clinical variables
Pace et al., 2010	Single group. Pre only	Same as Pace et al. (2009)	University students, *N* = 30 (40% female) No mediation experience	(1) **[Beh/Bio]** Group level comparison is not applicable	Measured by number of sessions per week, details were not available	**Significant Covariance:** Age and practice time were significantly correlated with amount of practice **Non-significant Covariance:** Cortisol, IL-6, and distress responses during TSST were not correlated with amount of practice
Pace et al., 2013	RCT: CBCT vs. waitlist control. Pre-Post-six-week FU	CBCT: 1-h classes twice per week for 6 weeks, 30 min of at-home practice with disk every day	Adolescents in foster care system. CBCT: *N* = 29 (mean age = 14.55; *SD* = 1.21; 44.83% female) Waitlist: *N* = 26 (Mean age = 14.96; *SD* = 1; 42.31% female) Unknown meditation experience	**Non-significant Effects:** (1) **[Bio]** No between-group difference in salivary C-reactive protein concentrations (2) No between-group difference in depression and anxiety	Times of meditation practice each day, collected weekly: 17.09 ± 20.69) times over course; increased from first 3 weeks (7.8 ± 8.33 min) to last 3 sessions (11.59 ± 16.37 min)	**Significant Covariance:** Practice sessions were correlated with reduced C-reactive protein **Non-significant Covariance:** Practice sessions were not correlated with baseline or post-intervention depression or anxiety
Reddy et al., 2013	Same as Pace et al. (2013)	Same as Pace et al. (2013)	Same as Pace et al. (2013)	**Non-significant Effects:** (1) No group difference in anxiety, depression, hope, attitudes to self and others, difficulty in emotion regulation, callous and unemotional traits (self-report and parent report)	See Pace et al., 2013	**Significant Covariance:** Practice frequency in last 3 weeks was correlated with increased hopefulness **Non-significant Covariance:** Total sessions and frequency in last 3 weeks were not correlated with other baseline or post-intervention psychosocial variables

***(A)** “CM” refers to compassion meditation. “Pre,” “Post,” and “FU” refer to pre-treatment measurement, post-treatment measurement, and follow up measurement, respectively. **(B)** This column sums all the outcomes measured in the studies; however, these results that are not relevant to the CM intervention (e.g., differences between two control groups) are omitted. Outcomes with significant results at any time point will no longer be listed as non-significant results at other time points. The results for conditions under the same concept are grouped (e.g., video with high and low emotion as an emotional video) and are considered significant if parts of those results are significant. **[Bio]**, **[Neu]**, and **[Beh]** refer to using behavioral tasks with dependent variables, biological or physiological indicators and neuro-imaging, respectively; other variables are self-reported daily variables. **(C)** This column only lists the effects of amount of practice that were reported in the articles; that is, those variables listed in group-level results (fourth column), but omitted here, are due to selective reporting*.

Jazaieri et al. (2013) evaluated Compassion Cultivation Training (CCT) with a wait-list control group in an RCT among community adults. The CCT lasted for 8 weeks and required 15 min of at-home practice every day. The recorded average formal meditation was 101.11 ± 57.00 min per week. The results showed that the CCT group had a significant increase in self-compassion and a decrease in fear of compassion (i.e., fear of compassion for others, for self, and from others). Time of meditation practice was significantly correlated with fear of compassion for others only. In another article on the same intervention (Jazaieri et al., 2014), the authors reported that the CCT group also showed improvements in mindfulness, decentering, worry, and expressive suppression, but not cognitive appraisal, happiness, and perceived stress. The time of formal meditation practice was significantly correlated with less worry and lower frequency of expressive suppression only. Furthermore, daily experience sampling was also conducted in the CCT group (Jazaieri et al., 2015), and the CCT significantly decreased general and specifically neutral mind wandering (but not positive and negative mind wandering) and significantly increased daily caring behavior for oneself (but not for others). The daily practice of meditation increased throughout the interventions and no sex differences were found. The amount of meditation increased caring behavior for oneself and others via increased positive mind wandering and decreased negative mind wandering as meditators.

While the above studies were concerned with daily outcomes, two interventions involved the immediate effects of compassion meditation after intervention. Leiberg et al. (2011) evaluated the effects of CM training compared with matched memory training in a non-RCT design among healthy female volunteers. The CM training consisted of a 1-day, 6-h session and participants had at least 2 h of at-home practice, although the details were not available. The participants were tested on the Zurich Prosocial Game (ZPG), which involved helping behavior with three pairs of experimental conditions: reciprocity (no reciprocity, reciprocity), cost (low, high), and distress (no distress, distress). The results showed that the CM group increased their helping behavior after the training, that their helping behaviors were significantly higher than those of a control group in both the high- and low-cost trials at post-training, and that the practice time of meditation at home was only positively associated with helping in no-reciprocity trials in the CM group. Furthermore, the CM group showed a decrease in negative mood and an increase in compassion. Both groups showed increases in positive moods, and neither group showed a change in the dictator game. No effect of practice time was explored for these variables. An additional memory task as a filler task was omitted here. Most participants also took part in fMRI scanning before and after interventions; these participants reported 5.7 h of meditation practice outside of the training (Klimecki et al., 2012). The task engaged in during the scan was called the “socio-affective video task” (SoVT): the participants viewed high-emotion videos that depicted others in distress or low-emotion videos where people performed daily activities. The participants were required to actively apply their skills after the intervention. When viewing the videos, the LKM group showed increased self-reported positive affect and empathy in comparison with the memory group. They also showed stronger brain activation related to positive affect and affiliation (mOFC, VTA/SN, putamen, and pallidum). However, no correlation between practice time and any outcome was found in this article, and the authors explained that the self-reported practice time might not be reliable.

The same research laboratory (Klimecki et al., 2013) conducted another non-RCT study among healthy female volunteers. One group (affective group) of participants first received 6 h of empathy training, with 98.6 ± 60.91 min of daily practice, and then received 6 h of CM training that was the same as above (Klimecki et al., 2012) after 5 days, with 65.72 ± 53.35 min of daily practice. Another group (memory group) that contained the same participants as the memory group in the previous study (Klimecki et al., 2012) attended a second memory training with a few detail adjustments. The task was also a SoVT task with an additional memory task that is omitted here. In brief, comparisons within the affective group confirmed that empathy training enhanced empathy, but also negative affect, while CM training decreased negative affect (to baseline) and increased positive affect during the video viewing. A series of increases in brain activation were reported after empathy training (anterior insula and anterior midcingulate cortex) and CM training (mOFC, pACC, IFG, and ventral striatum). However, no correlations between practice time and any behavioral or neural results were found.

### Other interventions

Neff and Germer (2013, study 2) developed a mindful self-compassion (MSC) program that focused on self-compassion. They compared MSC with a wait-list control group in an RCT among community adults. Notably, 78% of participants had previous meditation experience (see Tables 2–4). The 8-week MSC required 40 min of various practice every day. It is reported that participants performed formal practice (including FIM and other practices) 5.48 ± 1.50 days per week, and informal practice 5.48 ± 5.95 times per day. In comparison with the wait-list control group, MSC practitioners showed greater improvements in self-compassion, mindfulness, compassion for others, life satisfaction, depression, anxiety, stress, and avoidance, but not in social connection and happiness. The researchers only calculated the relationship between amount of practice and change in self-compassion, and the results showed that both formal practice and informal practice were positively correlated with a change in self-compassion.

**Table 4:** **Studies on other interventions based on four immeasurables meditations**.

**Study**	**Design (A)**	**Intervention**	**Participants**	**Results on group level (B)**	**Amount of meditation practice**	**Effect of meditation practice**
Neff and Germer, 2013	RCT: Mindful Self-compassion (MSC) vs. wait-list control. Pre-Post-6-month FU-one-year FU	MSC: participants attended 8 2-h weekly meetings, 40 min of practice every day	Community adults MSC: *N* = 24 (Mean age = 51.21; *SD* = 12.02; 78% female), Control: *N* = 27 (Mean age = 49.11; *SD* = 11.59; 82% female) 78% had meditation experience	**Positive Results:** (1) MSC group showed greater improvement in self-compassion, mindfulness, compassion for others, life satisfaction, depression, anxiety, stress, and avoidance than control group **Non-significant Results:** (1) No significant group differences for social connection and happiness	Number of days per week of practicing formal meditations (not only compassion meditation), number of times per day practicing informal self-compassion practice: 5.48 ± 1.50 days per week with formal practice, 5.48 ± 5.95 times per day for informal practice	**Significant Covariance:** Increases in self-compassion were significantly related to number of days per week that participants meditated and number of times per day that they informally practiced Authors confirmed that only self-compassion was concerned and calculated
Wallmark et al., 2013	RCT: Four Immeasurables Intervention (FII) vs. wait-list control group. Pre-Post	Weekly 75-min class for 8 weeks, with 30 min of practice at home every day	Healthy community adults, FII: *N* = 20 (Mean age = 32; *SD* = 11; 86% female) Control: *N* = 22 (Mean age = 35; *SD* = 15; 86% female) No meditation experience	**Positive Effects:** (1) Stress, perspective taking, mindfulness (except non-judge) and self-compassion in comparison with control at post intervention (2) Altruistic-oriented tendency increase in FII group only **Non-significant Results:** (1) Personal distress, empathic concern	Amount of meditation practice in minutes: 1234.3 ± 342.5 (via personal communication)	**Significant Covariance:** Amount of practice was correlated with decrease in perceived stress, increase in mindfulness (total score but not dimensions), and altruistic orientation Non-significant Covariance: Amount of practice was not correlated with change in self-compassion (but dimension of mindfulness) and dimensions in trait of empathy

***(A)** “Pre,” “Post,” and “FU” refer to pre-treatment measurement, post-treatment measurement, and follow up measurement, respectively. **(B)** This column sums all the outcomes measured in the studies. Outcomes with significant results at any time point will no longer be listed as non-significant results at other time points. The results of sub-dimensions of concepts are grouped (e.g., mindfulness) and are considered significant if parts of those results are significant. All variables are self-reported daily variables.*

Wallmark et al. (2013) developed an 8-week “four immeasurables intervention” that covered all four FIM subtypes. They compared this intervention with a wait-list control group in an RCT among healthy community adults. In comparison with the control group at post intervention, the intervention group reported significantly lower perceived stress and greater mindfulness (except for the dimension of “non-judge”), self-compassion, and perspective taking; however, no significant differences were found in personal distress and empathic concern. Additionally, the altruism-oriented tendency also increased in the intervention group, but not in the control group. Among all variables, total practice time was significantly correlated with an increase in mindfulness (total score) and the altruism-oriented tendency, and a reduction in perceived stress.

## Discussion

### Studies were few and varied in many aspects

Among the articles identified by systematic literature search, only 20 out of 51 (39%) articles on FIM interventions evaluated the relationship between amount of meditation practice and dependent (and/or independent) variables, which reflects the fact that current studies on amount of meditation practice or more generally, on active components, are still limited. Among them, four studies (Carson et al., 2005; Leiberg et al., 2011; May et al., 2011; Neff and Germer, 2013) calculated the relationship between amount of practice and parts of outcomes rather than all reported outcomes. Neff and Germer (2013) mentioned that they only calculated these relationships for variables that were of primary concern; others did not report the reasons for their chosen calculations. Similarly, another 13 articles (25.5%) noted that they had recorded the amount of practice, but did not further calculate its relationship with outcomes (see “Results of literature search” section). Ultimately, the current data on the effects of meditation practice are few, and future studies should explore the effects of meditation on outcomes.

The reviewed studies showed large heterogeneity. This review organized studies into LKM, CM, and other interventions; however, no consistent pattern for LKM or CM, or obvious differences between them, were observed. The interventions' contents in each category were also different, and only CBCT participants experienced repeated application. Of note, among all identified articles on FIM interventions, no intervention used AJM or EM as primary meditation practice. Currently, the only empirical study on AJM was an experiment in a laboratory that evaluated the effects of 6 min AJM practice (Zeng et al., 2017). Moreover, a focused study on the concept of appreciative joy is also at its very beginning (Zeng et al., 2016). As far as we know, no empirical study on EM is available now. Additionally, it is only very recently that scholars began to differentiate FIM in empirical studies (Zeng et al., 2017). Consequently, this review cannot include AJM and EM due to a lack of data.

Regarding outcome variables, among all 22 reviewed studies, 16 studies adopted various self-reported scales to measure daily outcomes, eight studies involved various behavioral tasks with dependent variables (i.e., TSST, RMET, EFP, SoVT, ZPG, dictator game, perception of pain, and attentional blink), five studies used biological or physiological indicators (i.e., IL-6; cortisol; C-reactive protein, ECG), and five studies measured brain activation in different tasks (see Tables 2–4). Among all variables, mindfulness (Fredrickson et al., 2008; Cohn and Fredrickson, 2010; May et al., 2011; Wallmark et al., 2013; Jazaieri et al., 2014), self-compassion (Neff and Germer, 2013; Wallmark et al., 2013; Jazaieri et al., 2014; Weibel et al., 2016), hope (Fredrickson et al., 2008; Cohn and Fredrickson, 2010; Reddy et al., 2013), depression (Fredrickson et al., 2008; Desbordes et al., 2012; Pace et al., 2013), anxiety (Carson et al., 2005; Weibel et al., 2016), empathy (Wallmark et al., 2013; Leppma and Young, 2016) and responses in SoVT (Klimecki et al., 2012, 2013) were evaluated as outcomes in more than one intervention (i.e., variables used in baseline measurement only or duplicated variables from articles based on the same intervention are omitted here), and the findings on mindfulness, self-compassion, and depression were in contradictory across studies. Additionally, other factors such as clinical and non-clinical samples in different studies may also have influenced the results. Therefore, it is difficult to draw solid conclusions for each intervention and each outcome.

### Contributions of meditation practice tend to be small

In general, the current review showed that there are relatively few significant associations between outcome variables and amount of meditation practice. Only five studies (Carson et al., 2005; Fredrickson et al., 2008; Pace et al., 2009; Neff and Germer, 2013; Jazaieri et al., 2015) reported that meditation practice was associated with more than half of the outcome variables. This included indirect relationships via mediation effects (Fredrickson et al., 2008; Jazaieri et al., 2015) and excluded variables that were not reported (Carson et al., 2005; Neff and Germer, 2013). The associations between meditation practice and outcome variables were minimal in other studies, or completely non-existent in seven studies (Pace et al., 2010; May et al., 2011; Desbordes et al., 2012; Mascaro et al., 2012; Klimecki et al., 2012, 2013; Weibel et al., 2016). From another perspective, most of the significant findings on amount of practice were accompanied by significant group effects (e.g., intervention group vs. control group), and only six studies noted that certain variables were not significant at the group level, but co-varied with amount of practice (Carson et al., 2005; Pace et al., 2009, 2013; Mascaro et al., 2013; Reddy et al., 2013; Dodds et al., 2015). In contrast, significant results for the effects of amount of practice were far fewer than the significant results at the group level. Such trends suggest that the contribution of meditation practice to the effects of FIM interventions is relatively small. From another perspective, only Cohn and Fredrickson (2010) were clearly concerned with whether the effects would remain if meditation practice stopped, while other studies did not record information on continuous practice after the intervention ended. The findings of Cohn and Fredrickson (2010) also supported the idea that continual meditation practice may not be necessary for maintenance of effects, which also raises questions about its contribution. Additionally, even without meditation, the loving-kindness discussion group can change attitudes toward the self (Kang et al., 2015), which showed that other components in FIM's interventions also contribute to the effects. In sum, the current findings imply that the contributions of meditation practice might be limited. Considering the costs of meditation practice, it is important to conduct more studies to investigate the necessary amount of meditation practice.

Additionally, as highlighted by the reviewers of current article, one might question what the meaning is when the whole intervention group is not effective (in comparison with control group), but effect of amount of meditation practice existed. Pace et al. (2009) noted that the group level difference and effect of amount of meditation practice could be detached in meditation studies; however, further investigation on such phenomena seems rare in reviewed studies. Since there must be variance among participants in intervention groups (thus the correlation is possible), we suggest scholars to further divide participants per amount of meditation practice (e.g., Pace et al., 2009) or any other variables measured before intervention. In doing so, scholars could explore whether the effect of the intervention is limited to participants with more meditation practice or other pre-existing individual differences. Of course, other explanations might also exist. For example, it is possible that the small sample size in intervention studies cannot provide enough power to detect group differences. Nevertheless, it is confusing to reveal a result where part of the intervention (i.e., meditation) is effective, but the whole intervention has no effect. Therefore, future studies should provide more explanation when the effect of amount of meditation practice exists but the effects of the whole intervention are not observed.

Another issue, pointed out by reviewers of current article, is that the length of reviewed interventions was no longer than 8 weekly sessions, which was far shorter than years of meditation practice in traditional Buddhist training. Therefore, researchers should be cautious when generalizing the conclusion. Furthermore, the limited duration of interventions also limit the variance in amount of meditation practice; therefore, it is possible that larger differences in meditation experience (e.g., 10000–50000 h in previous studies; Lutz et al., 2004) may result in more observable differences.

### Short-term influences have been less explored but are more promising

The reviewed studies tended to use the total amount of meditation practice during the whole intervention, rather than the amount of practice in the short term (e.g., during the most recent week), to predict the outcomes. Only four studies measured daily outcomes and explored their relationship with daily meditation practice (Carson et al., 2005; Fredrickson et al., 2008; Cohn and Fredrickson, 2010; Jazaieri et al., 2015). Pace et al. (2009) selected meditation practice in the last 3 weeks, and Weibel et al. (2016) reported meditation practice over the prior week, which could also be considered a concern for short-term effects. In contrast, all other studies summarized the total amount of meditation practice, although most collected data weekly or even daily. Although the number of studies that measured the frequency of practice was few, it is notable that the frequency of practice was significantly associated with more than half of the calculated variables in all articles except Weibel et al. (2016). In contrast, studies that calculated the total amount of meditation found relatively fewer significant effects. Such results imply that short-term influences are promising.

It is notable that no study explicitly explained why it calculated total amount of meditation or the frequency of short-term practice. It seems that most researchers held the (implicit) assumption that meditation practice has its effects in a cumulative way through long-term practice, while few researchers explored a pattern of short-term change. In addition, although most Buddhist traditions encourage long-term meditation practice, Zen Buddhism claims a potential “sudden change,” which asserts that the enlightenment can occur with an instant revelation (Suzuki, 1991). Although the enlightenment in Zen Buddhism is not necessarily the cultivation of four immeasurables (Suzuki, 1991), such pattern of “sudden change” is also reasonable in FIM: FIM invites people to experience positive emotions during blessings for others (Sujiva, 2007), and this experience may enable practitioners to build the belief that it feels good to help others and so on. Such changes in belief or knowledge may be achieved in meditation experience once and forever, without the necessity of further meditation practice. In all, future studies could explore different patterns of change, and a more explicit theoretical prediction on how this change happens would benefit the research.

### The mechanism from meditation practice to daily outcomes needs more exploration

How meditation practice leads to changes in daily life was not well explained. For example, Fredrickson et al. (2008) identified a pathway from meditation to positive emotions, resources, and finally to ultimate outcomes; however, this study did not answer the question of how LKM practice initially enhances daily positive emotions. Notably, the daily positive emotions in this study included a wide range, including pride. However, a study on one-shot practice of LKM (Hutcherson et al., 2015) reported that LKM did not generate pride. Therefore, there must be another mechanism connecting the amount of meditation practice to a wide range of positive emotions in daily life. Similarly, Neff and Germer (2013) found that the amount of meditation was associated with self-compassion, but how the repeated practice benefits self-compassion is unknown. Although positive emotions and positive attitudes (including self-compassion) are directly targeted and cultivated in FIM (Zeng et al., 2015), researchers still need to determine how and why these variables are influenced by meditation. As for outcomes that were indirectly targeted by FIM (e.g., decreased stress reactions; Pace et al., 2009), a more underlying mechanism needs to be clarified. Notably, reviewers of current article also pointed out that most of the reviewed studies did not include four immeasurables as outcomes variables, although the four immeasurables should be the primary outcomes of FIM and could be potential mediators for other outcomes.

From meditation practice to daily outcomes, one of the potential mechanisms is that repeated meditation practice makes practitioners more effective in an active application of FIM. Therefore, this enhances the effectiveness of emotional regulation when this is needed by practitioners. For example, previous studies showed that active application of CM when seeing the suffering of others generated positive feelings, which could regulate practitioners' emotions and let them approach and help suffering people (Klimecki et al., 2012, 2013; Weng et al., 2013). In such a case, one can expect that repeated practice of FIM makes it easier for practitioners to generate positive feelings when they need to. Two reviewed studies (Klimecki et al., 2012, 2013) evaluated active application of this CM skill. They did not find associations between amount of meditation practice and effects; however, the amount of meditation practice in these studies was small, and their authors noted that the record of meditation practice may not be reliable (Klimecki et al., 2013). In addition to interventions, some cross-sectional studies reported that experienced meditators had larger effects in measured outcomes during active FIM practice when compared to novices (e.g., Lutz et al., 2004), which also suggested the effects of repeated practice. However, cross-sectional studies are different in nature from interventions; therefore, such studies are not illustrated here. In all, future studies could pay more attention to ability in active application of FIM skills, which may link meditation practice in an intervention to daily outcomes. Furthermore, the exploration on active application of FIM skill also provides additional perspective to understand the effect of meditation practice. That is, the long-term meditation practice might change the implicit or potential abilities (it can be applied when needed), although such changes might not be explicitly reflected in daily outcomes.

Another mechanism for further investigation is that daily FIM practice affects short-term mental status, such as by inducing positive moods, and this leads to further change in daily life. This mechanism is more consistent with those studies that explored the short-term associations between amount of practice and outcomes as discussed above. Current studies indicated that FIM practice on a given day can influence one's life the same morning (Fredrickson et al., 2008), same day, or the next day (e.g., Carson et al., 2005); although, how long these short-term effects last is not yet clear. Similarly, studies on the one-shot practice of FIM in laboratory settings explored the immediate influence of FIM practice on emotions (e.g., Hutcherson et al., 2008), attitudes (e.g., Hutcherson et al., 2008), attention (e.g., Burgard and May, 2010), memory (Wheeler and Lenick, 2014), self-referential processing (Logie and Frewen, 2015), and so on. However, assessments in these studies were made no longer than 20 min after meditation practice, and it is also unknown how long these immediate effects could last. Notably, some variables such as positive emotions are believed to be transient, and there are also theories like the “broaden-and-build” theory that try to explain how positive emotions lead to further change (Fredrickson, 2001). In all, future studies could further explore how long immediate effects of FIM could last, or how such effects further impact daily life. With better understanding of these issues, researchers could better predict whether long term continuous meditation practice is necessary for certain outcomes.

### Confounding factors and methodological issues

The importance of meditation practice was supported by the correlations between the amount of meditation practice and outcomes; however, it is worth noting that this result may be confounded by other factors. The fundamental issue here is that the relationship between amount of meditation and effects in the reviewed studies is essentially correlational. That is, the researchers did not manipulate the amount of meditation practice into high vs. low levels, thus the causal relationships between meditation practice and outcomes cannot be confirmed. Fredrickson et al. (2008) and Mascaro et al. (2012) found that pre-existing individual differences could influence the amount of meditation practice during interventions. Even if there is no observed influence from baseline differences, it is possible that some practitioners were willing to practice more meditation because they experienced positive changes, had higher expectations, or endorsed some aspect of the interventions. Previous studies have already compared groups with and without meditation practice (e.g., Kang et al., 2015), and future studies could manipulate the amount or organization of meditation practice.

At the same time, methodological issues can obscure the relationship between meditation practice and effects, even when this relationship exists. The low reliability of meditation practice records has been noted above. Researchers also noted the possibility that the quality of practice is more important than the amount of practice (Shapiro et al., 2007; Zeng et al., 2015), and the quality of practice may also be influenced by many factors like individual difference of practitioners or experience of the trainers, as noted by reviewers of current article. Such possibilities could have reduced the association between amount of meditation practice and outcome changes. In sum, a better evaluation of amount and quality of meditation practice may benefit future studies. Table 5 summarizes the suggestions for future studies.

**Table 5:** **Potential methodological improvements and theoretical considerations in future studies**.

**Methodological suggestions**	Using objective records (e.g., electronic recorder) rather than subjective report to count amount of practice.
	Using daily or weekly report rather than recalling after the whole intervention.
	Recording both formal and informal meditation practice.
	Separating different meditation practice (e.g., different FIM).
	Measuring not only the time or quantity, but also quality of meditation practice.
	Figuring out time of meditation experience (i.e., concentration in meditation) rather than time spent on whole meditation practice.
**Theoretical considerations**	Considering the nature of outcomes and explaining why certain outcome should be impacted by meditation practice (or by other components in interventions like didactic components).
	Considering the nature of outcomes and predicting why meditation should have short term effect or long term effect on certain outcome.
	Investigating how meditation influences daily outcomes, especially for those outcomes that are not directly targeted in meditations (e.g., perceived stress)?
	Measuring not only effects on daily outcomes, but also effects on active application of meditation skill.

### Limitations

Two major limitations of the current review should be noted. First, this review was limited to articles published in journals; other resources, such as dissertations, might add more information. However, we believe the published articles represent the current paradigm in investigating the effects of meditation practice, and our summary of different perspectives and methodological issues should not have been heavily impacted by missing unpublished studies. Second, we discussed the issue of selective reporting, but did not provide a comprehensive risk of bias assessment. This is because the current review focused on associations between amount of meditation practice and outcome variables, which is largely limited to FIM intervention groups and was less concerned with typical risks of bias such as randomization between groups or allocation concealment.

## Conclusions

Whether more meditation practice leads to better outcomes in FIM interventions has received insufficient research attention. Current evidence shows that the amount of meditation practice has limited associations with outcomes. In addition to correlations between total amount of practice and daily outcomes, the short-term influence of FIM and the ability of actively applying FIM skills present promising perspectives to understand the contribution of meditation practice. Experimental manipulation and better measurement of meditation practice are needed in future studies.

## Author contributions

XZ, FL, and XL designed the study. XZ and FC reviewed articles and analyzed the data. XZ and TO wrote the paper. All authors discussed the results and commented on the manuscript.

### Conflict of interest statement

The authors declare that the research was conducted in the absence of any commercial or financial relationships that could be construed as a potential conflict of interest.
