# Supplementary material for: A Systematic Review of Associations between Amount of Meditation Practice and Outcomes in Interventions Using the Four Immeasurables Meditations
Source: Front Psychol. 2017 Feb 6;8:141. doi: 10.3389/fpsyg.2017.00141 (PMC5292580; doi:10.3389/fpsyg.2017.00141)
Supplement: Supplementary file 1 [file Table1.PDF]

Supplementary Material

A systematic review of associations between amount of meditation practice and outcomes in interventions using the Four Immeasurables Meditations

Xianglong ZENG, Floria HN CHIO, Tian PS. OEI, Freedom YK. LEUNG, Xiangping LIU*

*** Correspondence:** Xiangping LIU: lxp599e@163.com

1 Supplementary Figures and Tables

1.1 Supplementary Tables

Supplementary Table 1. List of identified empirical studies on FIMs

Studies	Type of studies	Information on Meditation Practice
Alba (2013).	FIM Intervention	Only recorded the amount of practice
Arch, Brown, Dean, Landy, Brown & Laudenslager (2014)	FIM Intervention	Not available
Barnhofer, Chittka, Nightingale, Visser & Crane (2010)	One-shot practice of FIM	Not Applicable
Boellinghaus, Jones & Hutton (2013)	FIM Intervention	Not available
Brewer, Worhunsky, Gray, Tang, Weber & Kober (2011)	FIM Meditators	Only recorded the meditation experience
Burgard & May (2010)	One-shot practice of FIM	Not Applicable
Carson, et al. (2005)	FIM Intervention	Reviewed, see main document
Cohn & Fredrickson (2010)	FIM Intervention	Reviewed, see main document
Condon, Desbordes, Miller & DeSteno (2013)	FIM Intervention	Only recorded the amount of practice
Crane, Jandric, Barnhofer & Williams (2010)	One-shot practice of FIM	Not Applicable
Desbordes, Negi, Pace, Wallace, Raison & Schwartz (2012)	FIM Intervention	Reviewed, see main document
Dodds et al. (2015)	FIM Intervention	Reviewed, see main document
Engen & Singer (2015)	FIM Meditators	Only recorded the meditation experience

Erdynast, & Rapgay (2009)	FIM Intervention	Only recorded the amount of practice
Feldman, Greeson & Senville (2010)	One-shot practice of FIM	Not Applicable
FeliuSoler et al. (2016)	FIM Intervention	Not available
Fredrickson, Cohn, Coffey, Pek & Finkel (2008)	FIM Intervention	Reviewed, see main document
Garrison, Scheinost, Constable & Brewer (2014)	FIM Meditators	Only recorded the meditation experience
He, Shi, Han, Wang, Zhang &Wang (2015)	FIM Intervention	Not available
Hofmann et al. (2015)	FIM Intervention	Not available
Hoge et al. (2013)	FIM Meditators	Only recorded the meditation experience
Hunsinger, Livingston & Isbell (2013)	FIM Intervention	Not available
Hunsinger, Livingston & Isbell (2014)	FIM Meditators	Only recorded the meditation experience
Hutcherson, Seppala & Gross (2008)	One-shot practice of FIM	Not Applicable
Hutcherson, Seppala & Gross (2015)	One-shot practice of FIM	Not Applicable
Jazaieri et al. (2013)	FIM Intervention	Reviewed, see main document
Jazaieri et al. (2014)	FIM Intervention	Reviewed, see main document
Jazaieri et al. (2015)	FIM Intervention	Reviewed, see main document
Johansson, Bjuhr & Rönnbäck (2015)	FIM Intervention	Not available
Johnson et al. (2009)	FIM Intervention	Not available
Johnson et al. (2011)	FIM Intervention	Only recorded the amount of practice
Kang, Gray & Dovidio (2013)	FIM Intervention	Only recorded the amount of practice
Kang, Gray & Dovidio (2014)	FIM Intervention	Not available
Kearney, Malte, McManus, Martinez, Felleman & Simpson (2013)	FIM Intervention	Not available
Kearney, McManus, Malte, Martinez, Felleman & Simpson (2014)	FIM Intervention	Not available
Kemper & Shaltout (2011)	One-shot practice of FIM	Not Applicable
Kemper, Powell, Helms & Kim-sharpiro (2014)	FIM Meditators	Only recorded the meditation experience

Klimecki, Leiberg, Lamm & Singer (2013)	FIM Intervention	Reviewed, see main document
Klimecki, Leiberg, Ricard & Singer (2014)	FIM Intervention	Reviewed, see main document
Kok et al. (2013)	FIM Intervention	Only recorded the amount of practice
Koopmann-Holm, Sze, Ochs & Tsai (2013)	FIM Intervention	Only recorded the amount of practice
Lee et al. (2012)	FIM Meditators	Only recorded the meditation experience
Leiberg, Klimecki & Singer (2011)	FIM Intervention	Reviewed, see main document
Leung, Chan, Yin, Lee, So, Lee (2012)	FIM Meditators	Reviewed, see main document
Leung, Chan, Yin, Lee, So, Lee (2015)	FIM Meditators	Only recorded the meditation experience
Logie & Frewen (2014)	One-shot practice of FIM	Not Applicable
Lumma, Kok & Singer (2015)	FIM Intervention	Not available
Lutz, Brefczynski-Lewis, Johnstone & Davidson (2008)	FIM Meditators	Reviewed, see main document
Lutz, Greischar, Perlman & Davidson (2009)	FIM Meditators	Only recorded the meditation experience
Lutz, Greischar, Rawlings, Ricard & Davidson (2004)	FIM Meditators	Only recorded the meditation experience
Mascaro, Rilling, Negi & Raison (2012)	FIM Intervention	Reviewed, see main document
Mascaro, Rilling, Negi & Raison (2013)	FIM Intervention	Reviewed, see main document
May et al. (2011)	FIM Intervention	Reviewed, see main document
May, Weyker, Spengel, Finkler & Hendrix (2014)	FIM Intervention	Only recorded the amount of practice
McCall, Steinbeis, Richard & Singer (2014)	FIM Meditators	Not available
Neff & Germer (2013)	FIM Intervention	Reviewed, see main document
Pace et al. (2009)	FIM Intervention	Reviewed, see main document
Pace et al. (2010)	FIM Intervention	Reviewed, see main document
Pace et al. (2013)	FIM Intervention	Reviewed, see main document
Pagliaro et al. (2016)	FIM Intervention	Not available
Parks, Birtel & Crisp (2014)	One-shot practice of FIM	Not Applicable
Pidgeon, Ford & Klaassen (2014)	FIM Intervention	Only recorded the amount of practice
Rana (2015)	FIM Intervention	Not available
Reddy et al. (2013)	FIM Intervention	Reviewed, see main document

Schutte (2014)	FIM Intervention	Only recorded the amount of practice
Sears & Kraus (2009)	FIM Intervention	Only recorded the amount of practice
Sears, Kraus, Carlough & Treat (2011)	FIM Intervention	Not available
Seppala, Hutcherson, Nguyen, Doty & Gross (2015)	One-shot practice of FIM	Not Applicable
Shahar et al. (2014)	FIM Intervention	Not available
Shaltout, Tooze, Rosenberger & Kemper (2012)	One-shot practice of FIM	Not Applicable
Stell & Farsides (2015)	One-shot practice of FIM	Not Applicable
Tonelli & Wachholtz (2012)	One-shot practice of FIM	Not Applicable
Wallmark, Safarzadeh, Daukantaitė & Maddux (2013)	FIM Intervention	Reviewed, see main document
Weng et al. (2013)	FIM Intervention	Only recorded the amount of practice
Weng, Fox, Hessenthaler, Stodola & Davidson (2016)	FIM Intervention	Only recorded the amount of practice
Weytens, Luminet, Verhofstadt & Mikolajczak (2014)	FIM Intervention	Not available
Wheeler & Lenick (2014)	One-shot practice of FIM	Not Applicable
Williams et al. (2005)	FIM Intervention	Not available

Supplementary Table 2. Articles that were not empirical studies on FIMs

Reason	Studies
**Empirical Study not relevant to FIMs**	Albertson, Neff & Dill-Shackleford (2015).
	Allen et al. (2012)
	Arimitsu & Hofmann (2015).
	Bach & Guse (2015).
	Baer, Lykins & Peters (2012).
	Bond, Mason, Lemaster, Shaw, Mullin, Holick & Saper (2013).
	Boyd-Wilson & Walkey (2015)
	Collinge, Kahn & Soltysik (2012).
	Colzato, Hommel, van den Wildenberg & Hsieh (2010).
	Colzato, Zech, Hommel, Verdonschot, van den Wildenberg & Hsieh (2012).
	Danucalov et al. (2013)
	Engstrom & Soderfeldt (2010).
	Flook, Goldberg, Pinger, Davidson (2015)
	Frewen, Rogers, Flodrowski & Lanius (2015)
	Greeson, Juberg, Maytan, James & Rogers (2014).
	Hinton, Pich, Hofmann & Otto (2013).
	Jennings, Frank, Snowberg, Coccia & Greenberg (2013).
	Judge, Cleghorn, McEwan & Gilbert (2012).
	Kelly, Zuroff, Foa & Gilbert (2010).
	Kemeny et al. (2012).
	Kemper, Bulla, Krueger, Ott, McCool & Gardiner (2011).
	Kemper, Shaltout, Tooze & Rosenberger (2012).
	Kim, Lee, Kim, Whang & Kang (2013).
	Kjellgren & Taylor (2008).
	Kozasa, Lacerda, Menezes, Wallace, Radvany, Mello & Sato (2015).
	Kraus & Sears (2009).
	Lau, Leung, Chan, Wong & Lee (2015)
	Levenson, Ekman & Ricard (2012).
	Lincoln, Hohenhaus & Hartmann (2013).
	Lo (2014).
	Lord (2013).
	Lucre & Corten (2013).
	Luders, Kurth, Mayer, Toga, Narr & Gaser (2012).
	Mantzios, & Wilson (2014).
	Moss, Wintering, Roggenkamp, Khalsa, Waldman, Monti & Newberg (2012).
	Neff & Pommier (2013).
	Oman, Thoresen, Hedberg (2010)
	Pace (2013).
	Pidgeon, Ford & Klaassen (2014).
	Pruitt & McCollum (2010).
	Rosenberg et al. (2015)
	Shapiro (1992)

	Skipper, O'Donovan, Conlon & Clough (2015)<br>Van Gordon, Shonin, Sumich, Sundin, Griffiths, Woods & Proeve (2014)<br>Xu et al. (2014)
**Not original empirical study that published on peer-reviewed journal**	Alawabdeh & Salem (2015).<br>Berger (2011)<br>Carson (2006).<br>Crane, Jandric, Barnhofer, Williams (2011)<br>Denny & Stevens (2011).<br>Desbordes, Negi, Pace, Wallace, Raison & Schwartz (2014).<br>Heathers, Brown, Coyne & Friedman (2015).<br>Hinton, Ojserkis, Jalal, Peou & Hofmann (2013).<br>Kemper & Shaltout (2012).<br>Kemper, Shaltout, Tooze & Rosenberger (2012).<br>Law (2012).<br>Negi, Pace, Wallace, Raison & Schwartz (2014).<br>Pace et al. (2012)

References

Alawabdeh, E. S. (2015). Compassion in mental health. Middle East Journal of Nursing, 9(1), 21-24 4p.

Alba, B. (2013). Loving-kindness meditation: A field study. Contemporary Buddhism, 14(2), 187-203. doi:10.1080/14639947.2013.832494

Albertson, E. R., Neff, K. D., & Dill-Shackleford, K. E. (2015). Self-compassion and body dissatisfaction in women: A randomized controlled trial of a brief meditation intervention. Mindfulness, 6(3), 444-454. doi:10.1007/s12671-014-0277-3

Allen, M., Dietz, M., Blair, K. S., van Beek, M., Rees, G., Vestergaard-Poulsen, P., . . . Roepstorff, A. (2012). Cognitive-affective neural plasticity following active-controlled mindfulness intervention. Journal of Neuroscience,32(44), 15601-15610. doi:10.1523/JNEUROSCI.2957-12.2012

Arch, J. J., Brown, K. W., Dean, D. J., Landy, L. N., Brown, K. D., & Laudenslager, M. L. (2014). Self-compassion training modulates alpha-amylase, heart rate variability, and subjective responses to social evaluative threat in women. Psychoneuroendocrinology, 42, 49-58. doi:10.1016/j.psyneuen.2013.12.018

Arimitsu, K., & Hofmann, S. G. (2015). Cognitions as mediators in the relationship between self-compassion and affect. Personality and Individual Differences, 74, 41-48. doi:10.1016/j.paid.2014.10.008

Bach, J. M., & Guse, T. (2015). The effect of contemplation and meditation on 'great compassion' on the psychological well-being of adolescents. Journal of Positive Psychology, 10(4), 359-369. doi:10.1080/17439760.2014.965268

Baer, R. A., Lykins, E. L. B., & Peters, J. R. (2012). Mindfulness and self-compassion as predictors of psychological wellbeing in long-term meditators and matched nonmeditators. Journal of Positive Psychology, 7(3), 230-238. doi:10.1080/17439760.2012.674548

Barnhofer, T., Chittka, T., Nightingale, H., Visser, C., & Crane, C. (Mar 2010). State effects of two forms of meditation on prefrontal EEG asymmetry in previously depressed individuals. Mindfulness, 1(1), 21-27.

Berger, R. E. (2011). Re: Effect of compassion meditation on neuroendocrine, innate immune and behavioral responses to psychosocial stress. Journal of Urology, 186(4), 1325-1326.

Boellinghaus, I., Jones, F. W., & Hutton, J. (Nov 2013). Cultivating self-care and compassion in psychological therapists in training: The experience of practicing loving-kindness meditation. Training and Education in Professional Psychology, 7(4), 267-277.

Bond, A. R., Mason, H. F., Lemaster, C. M., Shaw, S. E., Mullin, C. S., Holick, E. A., & Saper, R. B. (2013). Embodied health: The effects of a mind-body course for medical students. Medical Education Online, 18, 20699. doi:10.3402/meo.v18i0.20699

Boyd-Wilson, B. M., & Walkey, F. H. (2015). The enlightenment scale: A measure of being at peace and open-hearted. Pastoral Psychology, 64(3), 311-325. doi:10.1007/s11089-013-0586-9

Brewer, J. A., Worhunsky, P. D., Gray, J. R., Tang, Y., Weber, J., & Kober, H. (2011). Meditation experience is associated with differences in default mode network activity and connectivity. Proceedings of the National Academy of Sciences of the United States of America, 108(50), 20254-20259. doi:10.1073/pnas.1112029108

Burgard, M. (2010). The effect of positive affect induction via metta meditation on the attentional blink. Journal of Articles in Support of the Null Hypothesis, 7(1), 8.

Carson JW, Keefe FJ, Lynch TR, Carson KM, Goli V, Fras AM, & Thorp SR. (2005). Loving-kindness meditation for chronic low back pain: Results from a pilot trial. Journal of Holistic Nursing, 23(3), 287-304.

Carson, J. W. (2006). Loving-kindness meditation findings not related to baseline differences. Journal of Holistic Nursing, 24(1), 5-6.

Cohn, M. A., & Fredrickson, B. L. (2010). In search of durable positive psychology interventions: Predictors and consequences of long-term positive behavior change. Journal of Positive Psychology, 5(5), 355-366. doi:10.1080/17439760.2010.508883

Collinge, W., Kahn, J., & Soltysik, R. (2012). Promoting reintegration of national guard veterans and their partners using a self-directed program of integrative therapies: A pilot study. Military Medicine, 177(12), 1477-1485. doi:10.7205/MILMED-D-12-00121

Colzato, L. S., Hommel, B., van den Wildenberg, W. P. M., & Hsieh, S. (2010). Buddha as an eye opener: A link between prosocial attitude and attentional control. Frontiers in Psychology, 1, 156. doi:10.3389/fpsyg.2010.00156

Colzato, L. S., Zech, H., Hommel, B., Verdonschot, R., van den Wildenberg, W. P. M., & Hsieh, S. (2012). Loving-kindness brings loving-kindness: The impact of buddhism on cognitive self-other integration. Psychonomic Bulletin & Review, 19(3), 541-545. doi:10.3758/s13423-012-0241-y

Condon, P., Desbordes, G., Miller, W. B., & DeSteno, D. (2013). Meditation increases compassionate responses to suffering. Psychological Science, 24(10), 2125-2127. doi:10.1177/0956797613485603

Contemplative/emotion training reduces negative emotional behavior and promotes prosocial responses. (2012). Emotion (Washington, D.C.), 12(2), 338-350.

Crane, C., Jandric, D., Barnhofer, T., & Williams, J. M. G. (Dec 2010). Dispositional mindfulness, meditation, and conditional goal setting. Mindfulness, 1(4), 204-214.

Crane, C., Jandric, D., Barnhofer, T., & Williams, J. M. G. (Jun 2011). Dispositional mindfulness, meditation, and conditional goal setting: Erratum. Mindfulness, 2(2), 142.

Danucalov, M. A. D., Kozasa, E. H., Ribas, K. T., Galduroz, J. C. F., Garcia, M. C., Verreschi, I. T. N., . . . Leite, J. R. (2013). A yoga and compassion meditation program reduces stress in familial

caregivers of alzheimer's disease patients. Evidence-Based Complementary and Alternative Medicine, , 513149. doi:10.1155/2013/513149

Denny, J., & Stevens, L. (2011.). EEG/loreta frequency and localization characteristics of compassion versus egocentrism versus universal mind. Journal of Neurotherapy, 15(4), 434.

Desbordes, G., Negi, L. T., Pace, T. W. W., Wallace, B. A., Raison, C. L., & Schwartz, E. L. (2014.). Effects of eight-week meditation training on hippocampal volume: A comparison of mindful attention training and cognitively-based compassion training. Journal of Alternative and Complementary Medicine, 20(5), A24.

Desbordes, G., Negi, L. T., Pace, T. W. W., Wallace, B. A., Raison, C. L., & Schwartz, E. L. (2012). Effects of mindful-attention and compassion meditation training on amygdala response to emotional stimuli in an ordinary, non-meditative state. Frontiers in Human Neuroscience, 6, 292. doi:10.3389/fnhum.2012.00292

Dodds, S., Pace, T. W. W., Bell, M., Fiero, M., Negi, L. T., Raison, C., & Weihs, K. (2015.). Feasibility and effects of cognitively-based compassion training (CBCT) on psychological well-being in breast cancer survivors: A randomized, wait list controlled pilot study. Psycho-Oncology, 24, 96-97.

Engen, H. G., & Singer, T. (Sep 2015). Compassion-based emotion regulation up-regulates experienced positive affect and associated neural networks. Social Cognitive and Affective Neuroscience, 10(9), 1291-1301.

Engstrom, M., & Soderfeldt, B. (2010). Brain activation during compassion meditation: A case study. Journal of Alternative and Complementary Medicine, 16(5), 597-599. doi:10.1089/acm.2009.0309

Erdynast, A., & Rapgay, L. (Mar 2009). Developmental levels of conceptions of compassion in the ethical decision-making of western buddhist practitioners. Journal of Adult Development, 16(1), 1-12.

Feldman, G., Greeson, J., & Senville, J. (Oct 2010). Differential effects of mindful breathing, progressive muscle relaxation, and loving-kindness meditation on decentering and negative reactions to repetitive thoughts.Behaviour Research and Therapy, 48(10), 1002-1011.

FeliuSoler, A., Pascual, J. C., Elices, M., MartinBlanco, A., Carmona, C., Cebolla, A., . . . Soler, J. (2016). Fostering self-compassion and loving-kindness in patients with borderline personality disorder: A randomized pilot study.Clinical Psychology & Psychotherapy, doi:http://dx.doi.org/10.1002/cpp.2000

Flook, L., Goldberg, S. B., Pinger, L., & Davidson, R. J. (2015). Promoting prosocial behavior and self-regulatory skills in preschool children through a mindfulness-based kindness curriculum. Developmental Psychology, 51(1), 44-51. doi:10.1037/a0038256

Fredrickson, B. L., Cohn, M. A., Coffey, K. A., Pek, J., & Finkel, S. M. (2008). Open hearts build lives: Positive emotions, induced through loving-kindness meditation, build consequential

personal resources. Journal of Personality and Social Psychology, 95(5), 1045-1062. doi:10.1037/a0013262

Frewen, P., Rogers, N., Flodrowski, L., & Lanius, R. (2015). Mindfulness and metta-based trauma therapy (mmtt): Initial development and proof-of-concept of an internet resource. Mindfulness,

Garrison, K. A., Scheinost, D., Constable, R. T., & Brewer, J. A. (2014). BOLD signal and functional connectivity associated with loving kindness meditation. Brain and Behavior, 4(3), 337-347. doi:10.1002/brb3.219

Greeson, J. M., Juberg, M. K., Maytan, M., James, K., & Rogers, H. (2014). A randomized controlled trial of koru: A mindfulness program for college students and other emerging adults. Journal of American College Health,62(4), 222-233. doi:10.1080/07448481.2014.887571

He, X., Shi, W., Han, X., Wang, N., Zhang, N., & Wang, X. (2015). The interventional effects of loving-kindness meditation on positive emotions and interpersonal interactions. Neuropsychiatric Disease and Treatment, 11, 1273-1277. doi:10.2147/NDT.S79607

Heathers, J. A. J., Brown, N. J. L., Coyne, J. C., & Friedman, H. L. (Jul 2015). The elusory upward spiral: A reanalysis of kok et al. (2013). Psychological Science, 26(7), 1140-1143.

Hinton, D. E., Ojserkis, R. A., Jalal, B., Peou, S., & Hofmann, S. G. (2013). Loving-kindness in the treatment of traumatized refugees and minority groups: A typology of mindfulness and the nodal network model of affect and affect regulation. Journal of Clinical Psychology, 69(8), 817-828. doi:10.1002/jclp.22017

Hinton, D. E., Pich, V., Hofmann, S. G., & Otto, M. W. (2013). Acceptance and mindfulness techniques as applied to refugee and ethnic minority populations with PTSD: Examples from "culturally adapted CBT". Cognitive and Behavioral Practice, 20(1), 33-46.

Hofmann, S. G., Petrocchi, N., Steinberg, J., Lin, M., Arimitsu, K., Kind, S., . . . Stangier, U. (2015). Loving-kindness meditation to target affect in mood disorders: A proof-of-concept study. Evidence-Based Complementary and Alternative Medicine, , 269126. doi:10.1155/2015/269126

Hoge, E. A., Chen, M. M., Orr, E., Metcalf, C. A., Fischer, L. E., Pollack, M. H., . . . Simon, N. M. (2013). Loving-kindness meditation practice associated with longer telomeres in women. Brain Behavior and Immunity, 32, 159-163. doi:10.1016/j.bbi.2013.04.005

Hunsinger, M., Livingston, R., & Isbell, L. (2013). The impact of loving-kindness meditation on affective learning and cognitive control. Mindfulness, 4(3), 275-280. doi:10.1007/s12671-012-0125-2

Hunsinger, M., Livingston, R., & Isbell, L. (2014). Spirituality and intergroup harmony: Meditation and racial prejudice. Mindfulness, 5(2), 139-144. doi:10.1007/s12671-012-0159-5

Hutcherson, C. A., Seppala, E. M., & Gross, J. J. (2008). Loving-kindness meditation increases social connectedness. Emotion, 8(5), 720-724. doi:10.1037/a0013237

Hutcherson, C. A., Seppala, E. M., & Gross, J. J. (2015). The neural correlates of social connection. Cognitive Affective & Behavioral Neuroscience, 15(1), 1-14. doi:10.3758/s13415-014-0304-9

Jazaieri, H., Jinpa, G. T., McGonigal, K., Rosenberg, E. L., Finkelstein, J., Simon-Thomas, E., . . . Goldin, P. R. (2013). Enhancing compassion: A randomized controlled trial of a compassion cultivation training program. Journal of Happiness Studies, 14(4), 1113-1126. doi:10.1007/s10902-012-9373-z

Jazaieri, H., Lee, I. A., McGonigal, K., Jinpa, T., Doty, J. R., Gross, J. J., & Goldin, P. R. (2016). A wandering mind is a less caring mind: Daily experience sampling during compassion meditation training. Journal of Positive Psychology, 11(1), 37-50. doi:10.1080/17439760.2015.1025418

Jazaieri, H., McGonigal, K., Jinpa, T., Doty, J. R., Gross, J. J., & Goldin, P. R. (2014). A randomized controlled trial of compassion cultivation training: Effects on mindfulness, affect, and emotion regulation. Motivation and Emotion, 38(1), 23-35. doi:10.1007/s11031-013-9368-z

Jennings, P. A., Frank, J. L., Snowberg, K. E., Coccia, M. A., & Greenberg, M. T. (2013). Improving classroom learning environments by cultivating awareness and resilience in education (CARE): Results of a randomized controlled trial. School Psychology Quarterly, 28(4), 374-390. doi:10.1037/spq0000035

Johansson, B., Bjuhr, H., & Ronnback, L. (Apr 2015). Evaluation of an advanced mindfulness program following a mindfulness-based stress reduction program for participants suffering from mental fatigue after acquired brain injury. Mindfulness, 6(2), 227-233.

Johnson, D. P., Penn, D. L., Fredrickson, B. L., Kring, A. M., Meyer, P. S., Catalino, L. I., & Brantley, M. (2011). A pilot study of loving-kindness meditation for the negative symptoms of schizophrenia. Schizophrenia Research,129(2-3), 137-140. doi:10.1016/j.schres.2011.02.015

Johnson, D. P., Penn, D. L., Fredrickson, B. L., Meyer, P. S., Kring, A. M., & Brantley, M. (2009). Loving-kindness meditation to enhance recovery from negative symptoms of schizophrenia. Journal of Clinical Psychology,65(5), 499-509. doi:10.1002/jclp.20591

Judge, L., Cleghorn, A., McEwan, K., & Gilbert, P. (2012). An exploration of group-based compassion focused therapy for a heterogeneous range of clients presenting to a community mental health team. International Journal of Cognitive Therapy, 5(4), 420-429.

Kang, Y., Gray, J. R., & Dovidio, J. F. (2014). The nondiscriminating heart: Lovingkindness meditation training decreases implicit intergroup bias. Journal of Experimental Psychology-General, 143(3), 1306-1313. doi:10.1037/a0034150

Kang, Y., Gray, J. R., & Dovidio, J. F. (2015). The head and the heart: Effects of understanding and experiencing lovingkindness on attitudes toward the self and others. Mindfulness, 6(5), 1063-1070. doi:10.1007/s12671-014-0355-6

Kearney DJ, McManus C, Malte CA, Martinez ME, Felleman B, & Simpson TL. (2014). Loving-kindness meditation and the broaden-and-build theory of positive emotions among veterans with posttraumatic stress disorder.Medical Care, 52(12 Suppl 5), S32-8.

Kearney, D. J., Malte, C. A., McManus, C., Martinez, M. E., Felleman, B., & Simpson, T. L. (2013). Loving-kindness meditation for posttraumatic stress disorder: A pilot study. Journal of Traumatic Stress, 26(4), 426-434. doi:10.1002/jts.21832

Kelly, A. C., Zuroff, D. C., Foa, C. L., & Gilbert, P. (2010). Who benefits from training in self-compassionate self-regulation? A study of smoking reduction. Journal of Social and Clinical Psychology, 29(7), 727-755.

Kemeny, M. E., Foltz, C., Cavanagh, J. F., Cullen, M., Giese-Davis, J., Jennings, P., . . . Ekman, P. (2012). Contemplative/Emotion training reduces negative emotional behavior and promotes prosocial responses. Emotion,12(2), 338-350. doi:10.1037/a0026118

Kemper, K., & Shaltout, H. (2012.). Non-verbal communication of compassion: Feasibility of measuring psychophysiological effects of blind exposure. BMC Complementary and Alternative Medicine, 12

Kemper, K., Shaltout, H., Tooze, J., & Rosenberger, E. (2012.). Time, touch, and compassion: Effects on autonomic nervous system and well-being. BMC Complementary and Alternative Medicine, 12

Kemper, K. J., Powell, D., Helms, C. C., & Kim-Shapiro, D. B. (2015). Loving-kindness meditation's effects on nitric oxide and perceived well-being: A pilot study in experienced and inexperienced meditators. Explore-the Journal of Science and Healing, 11(1), 32-39. doi:10.1016/j.explore.2014.10.002

Kemper, K. J., & Shaltout, H. A. (2011). Non-verbal communication of compassion: Measuring psychophysiologic effects. Bmc Complementary and Alternative Medicine, 11, 132. doi:10.1186/1472-6882-11-132

Kemper, K., Bulla, S., Krueger, D., Ott, M. J., McCool, J. A., & Gardiner, P. (2011). Nurses' experiences, expectations, and preferences for mind-body practices to reduce stress. Bmc Complementary and Alternative Medicine,11, 26. doi:10.1186/1472-6882-11-26

Kim, D., Lee, K., Kim, J., Whang, M., & Kang, S. W. (2013). Dynamic correlations between heart and brain rhythm during autogenic meditation. Frontiers in Human Neuroscience, 7, 414. doi:10.3389/fnhum.2013.00414

Kjellgren, A., & Taylor, S. (2008). Mapping zazen meditation as a developmental process: Exploring the experiences of experienced and inexperienced meditators. Journal of Transpersonal Psychology, 40(2), 224-250.

Klimecki, O. M., Leiberg, S., Lamm, C., & Singer, T. (2013). Functional neural plasticity and associated changes in positive affect after compassion training. Cerebral Cortex, 23(7), 1552-1561. doi:10.1093/cercor/bhs142

Klimecki, O. M., Leiberg, S., Ricard, M., & Singer, T. (2014). Differential pattern of functional brain plasticity after compassion and empathy training. Social Cognitive and Affective Neuroscience, 9(6), 873-879. doi:10.1093/scan/nst060

Kok, B. E., Coffey, K. A., Cohn, M. A., Catalino, L. I., Vacharkulksemsuk, T., Algoe, S. B., . . . Fredrickson, B. L. (2013). How positive emotions build physical health: Perceived positive social connections account for the upward spiral between positive emotions and vagal tone. Psychological Science, 24(7), 1123-1132. doi:10.1177/0956797612470827

Koopmann-Holm, B., Sze, J., Ochs, C., & Tsai, J. L. (2013). Buddhist-inspired meditation increases the value of calm. Emotion, 13(3), 497-505. doi:10.1037/a0031070

Kozasa, E. H., Lacerda, S. S., Menezes, C., Wallace, B. A., Radvany, J., Mello, Luiz E. A. M, & Sato, J. R. (2015). Effects of a 9-day shamatha buddhist meditation retreat on attention, mindfulness and self-compassion in participants with a broad range of meditation experience. Mindfulness,

Kraus, S., & Sears, S. (2009). Measuring the immeasurables: Development and initial validation of the self-other four immeasurables (SOFI) scale based on buddhist teachings on loving kindness, compassion, joy, and equanimity. Social Indicators Research, 92(1), 169-181. doi:10.1007/s11205-008-9300-1

Lau, W. K. W., Leung, M., Chan, C. C. H., Wong, S. S. Y., & Lee, T. M. C. (2015). Can the neural-cortisol association be moderated by experience-induced changes in awareness?. Scientific Reports, 5, 16620. doi:http://dx.doi.org/10.1038/srep16620

Law, R. W. (2012). An analogue study of loving-kindness meditation as a buffer against social stress. Dissertation Abstracts International: Section b: The Sciences and Engineering, 72(7-B), 4365.

Lee, T. M. C., Leung, M., Hou, W., Tang, J. C. Y., Yin, J., So, K., . . . Chan, C. C. H. (2012). Distinct neural activity associated with focused-attention meditation and loving-kindness meditation. Plos One, 7(8), e40054. doi:10.1371/journal.pone.0040054

Leiberg, S., Klimecki, O., & Singer, T. (2011). Short-term compassion training increases prosocial behavior in a newly developed prosocial game. Plos One, 6(3), e17798. doi:10.1371/journal.pone.0017798

Leung, M., Chan, C. C. H., Yin, J., Lee, C., So, K., & Lee, T. M. C. (2013). Increased gray matter volume in the right angular and posterior parahippocampal gyri in loving-kindness meditators. Social Cognitive and Affective Neuroscience, 8(1), 34-39. doi:10.1093/scan/nss076

Leung, M., Chan, C. C. H., Yin, J., Lee, C., So, K., & Lee, T. M. C. (2015). Enhanced amygdala-cortical functional connectivity in meditators. Neuroscience Letters, 590, 106-110. doi:10.1016/j.neulet.2015.01.052

Levenson, R. W., Ekman, P., & Ricard, M. (2012). Meditation and the startle response: A case study. Emotion, 12(3), 650-658. doi:10.1037/a0027472

Lincoln, T. M., Hohenhaus, F., & Hartmann, M. (2013). Can paranoid thoughts be reduced by targeting negative emotions and self-esteem? an experimental investigation of a brief compassion-focused intervention. Cognitive Therapy and Research, 37(2), 390-402. doi:10.1007/s10608-012-9470-7

Lo Herman, H. M. (2014). Applications of buddhist compassion practices among people suffering from depression and anxiety in confucian societies in east asia. J Br Music Ther, 33(1), 19-32.

Logie, K., & Frewen, P. (2015). Self/Other referential processing following mindfulness and loving-kindness meditation. Mindfulness, 6(4), 778-787. doi:10.1007/s12671-014-0317-z

Lord, S. A. (2013). Meditative dialogue: Cultivating compassion and empathy with survivors of complex childhood trauma. Journal of Aggression Maltreatment & Trauma, 22(9), 997-1014. doi:10.1080/10926771.2013.834018

Lucre, K. M., & Corten, N. (2013). An exploration of group compassion-focused therapy for personality disorder. Psychology and Psychotherapy-Theory Research and Practice, 86(4), 387-400. doi:10.1111/j.2044-8341.2012.02068.x

Luders, E., Kurth, F., Mayer, E. A., Toga, A. W., Narr, K. L., & Gaser, C. (2012). The unique brainan atomy of meditation practitioners: Alterations in cortical gyrification. Frontiers in Human Neuroscience, 6, 34. doi:10.3389/fnhum.2012.00034

Lumma, A., Kok, B. E., & Singer, T. (2015). Is meditation always relaxing? investigating heart rate, heart rate variability, experienced effort and likeability during training of three types of meditation. International Journal of Psychophysiology, 97(1), 38-45. doi:10.1016/j.ijpsycho.2015.04.017

Lutz, A., Greischar, L. L., Rawlings, N. B., Ricard, M. & Davidson, R. J. (2004). Long-term meditators self-induce high-amplitude gamma synchrony during mental practice. Proceedings of the National Academy of Sciences of the United States of America, 101(46), 16369-16373. doi:10.1073/pnas.0407401101

Lutz, A., Brefczynski-Lewis, J., Johnstone, T., & Davidson, R. J. (2008). Regulation of the neural circuitry of emotion by compassion meditation: Effects of meditative expertise. Plos One, 3(3), e1897. doi:10.1371/journal.pone.0001897

Lutz, A., Greischar, L. L., Perlman, D. M., & Davidson, R. J. (2009). BOLD signal in insula is differentially related to cardiac function during compassion meditation in experts vs. novices. Neuroimage, 47(3), 1038-1046. doi:10.1016/j.neuroimage.2009.04.081

Mantzios, M., & Wilson, J. C. (Apr 2014). Making concrete construals mindful: A novel approach for developing mindfulness and self-compassion to assist weight loss. Psychology & Health, 29(4), 422-441.

Mantzios, M., & Wilson, J. C. (2015). Exploring mindfulness and mindfulness with self-compassion-centered interventions to assist weight loss: Theoretical considerations and preliminary results of a randomized pilot study.Mindfulness, 6(4), 824-835. doi:10.1007/s12671-014-0325-z

Mascaro, J. S., Rilling, J. K., Negi, L. T., & Raison, C. L. (2013). Compassion meditation enhances empathic accuracy and related neural activity. Social Cognitive and Affective Neuroscience, 8(1), 48-55. doi:10.1093/scan/nss095

Mascaro, J. S., Rilling, J. K., Negi, L. T., & Raison, C. L. (2013). Pre-existing brain function predicts subsequent practice of mindfulness and compassion meditation. Neuroimage, 69, 35-42. doi:10.1016/j.neuroimage.2012.12.021

May, C. J., Burgard, M., Mena, M., Abbasi, I., Bernhardt, N., Clemens, S., . . . Williamson, R. (Sep 2011). Short-term training in loving-kindness meditation produces a state, but not a trait, alteration of attention.Mindfulness, 2(3), 143-153.

May, C. J., Weyker, J. R., Spengel, S. K., Finkler, L. J., & Hendrix, S. E. (2014). Tracking longitudinal changes in affect and mindfulness caused by concentration and loving-kindness meditation with hierarchical linear modeling. Mindfulness, 5(3), 249-258. doi:10.1007/s12671-012-0172-8

McCall, C., Steinbeis, N., Ricard, M., & Singer, T. (2014). Compassion meditators show less anger, less punishment and more compensation of victims in response to fairness violations. Frontiers in Behavioral Neuroscience,8, 424. doi:10.3389/fnbeh.2014.00424

Moss, A. S., Wintering, N., Roggenkamp, H., Khalsa, D. S., Waldman, M. R., Monti, D., & Newberg, A. B. (2012). Effects of an 8-week meditation program on mood and anxiety in patients with memory loss. Journal of Alternative and Complementary Medicine, 18(1), 48-53. doi:10.1089/acm.2011.0051

Neff, K. D., & Germer, C. K. (2013). A pilot study and randomized controlled trial of the mindful self-compassion program. Journal of Clinical Psychology, 69(1), 28-44. doi:10.1002/jclp.21923

Neff, K. D., & Pommier, E. (2013). The relationship between self-compassion and other-focused concern among college undergraduates, community adults, and practicing meditators. Self and Identity, 12(2), 160-176. doi:10.1080/15298868.2011.649546

Negi, L. T., Pace, T. W. W., Wallace Raison, C. L., & Schwartz, E. L. (2014). Effects of eight-week meditation training on hippocampal volume: A comparison of mindful attention training and cognitively-based compassion training. Journal of Alternative and Complementary Medicine (New York, N.Y.), 20(5), A24.

Oman, D., Thoresen, C. E., & Hedberg, J. (Mar 2010). Does passage meditation foster compassionate love among health professionals?: A randomised trial. Mental Health, Religion & Culture, 13(2), 129-154.

Pace, S. (2013). Does religion affect the materialism of consumers? an empirical investigation of buddhist ethics and the resistance of the self. Journal of Business Ethics, 112(1), 25-46. doi:10.1007/s10551-012-1228-3

Pace, T., Negi, L., Donaldson-Lavelle, B., Ozawa-de Silva, B., Reddy, S., Cole, S., . . . Raison, C. (2012.). Cognitively-based compassion training reduces peripheral inflammation in adolescents in foster care with high rates of early life adversity. BMC Complementary and Alternative Medicine, 12

Pace, T. W. W., Negi, L. T., Adame, D. D., Cole, S. P., Sivilli, T. I., Brown, T. D., . . . Raison, C. L. (2009). Effect of compassion meditation on neuroendocrine, innate immune and behavioral

responses to psychosocial stress.Psychoneuroendocrinology, 34(1), 87-98. doi:10.1016/j.psyneuen.2008.08.011

Pace, T. W. W., Negi, L. T., Dodson-Lavelle, B., Ozawa-de Silva, B., Reddy, S. D., Cole, S. P., . . . Raison, C. L. (2013). Engagement with cognitively-based compassion training is associated with reduced salivary C-reactive protein from before to after training in foster care program adolescents. Psychoneuroendocrinology, 38(2), 294-299. doi:10.1016/j.psyneuen.2012.05.019

Pace, T. W. W., Negi, L. T., Sivilli, T. I., Issa, M. J., Cole, S. P., Adame, D. D., & Raison, C. L. (2010). Innate immune, neuroendocrine and behavioral responses to psychosocial stress do not predict subsequent compassion meditation practice time. Psychoneuroendocrinology, 35(2), 310-315. doi:10.1016/j.psyneuen.2009.06.008

Pagliaro, G., Pandolfi, P., Collina, N., Frezza, G., Brandes, A., Galli, M., . . . Marconi, L. (2016). A randomized controlled trial of tong len meditation practice in cancer patients: Evaluation of a distant psychological healing effect. Explore: The Journal of Science & Healing, 12(1), 42-49. doi:http://dx.doi.org/10.1016/j.explore.2015.10.001

Parks, S., Birtel, M. D., & Crisp, R. J. (2014). Evidence that a brief meditation exercise can reduce prejudice toward homeless people. Social Psychology, 45(6), 458-465. doi:10.1027/1864-9335/a000212

Pidgeon, A. M., Ford, L., & Klaassen, F. (2014). Evaluating the effectiveness of enhancing resilience in human service professionals using a retreat-based mindfulness with metta training program: A randomised control trial.Psychology Health & Medicine, 19(3), 355-364. doi:10.1080/13548506.2013.806815

Pruitt, I. T., & McCollum, E. E. (Jun 2010). Voices of experienced meditators: The impact of meditation practice on intimate relationships. Contemporary Family Therapy: An International Journal, 32(2), 135-154.

Reddy, S. D., Negi, L. T., Dodson-Lavelle, B., Ozawa-de Silva, B., Pace, T. W. W., Cole, S. P., . . . Craighead, L. W. (2013). Cognitive-based compassion training: A promising prevention strategy for at-risk adolescents. Journal of Child and Family Studies, 22(2), 219-230. doi:10.1007/s10826-012-9571-7

Rosenberg, E. L., Zanesco, A. P., King, B. G., Aichele, S. R., Jacobs, T. L., Bridwell, D. A., . . . Saron, C. D. (2015). Intensive meditation training influences emotional responses to suffering. Emotion,

Schutte, N. S. (2014). The broaden and build process: Positive affect, ratio of positive to negative affect and general self-efficacy. Journal of Positive Psychology, 9(1), 66-74. doi:10.1080/17439760.2013.841280

Sears, S., Kraus, K., Carlough, E., & Treat. (2011). Perceived benefits and doubts of participants in a weekly meditation study. Mindfulness, 2(3), 167-174. doi:10.1007/s12671-011-0055-4

Sears, S., & Kraus, S. (2009). I think therefore I om: Cognitive distortions and coping style as mediators for the effects of mindfulness meditation on anxiety, positive and negative affect, and hope. Journal of Clinical Psychology, 65(6), 561-573. doi:10.1002/jclp.20543

Seppala, E., Seppala, C., Hutcherson, D., Nguyen, J., Doty, J., & Gross. (2014). Loving-kindness meditation: A tool to improve healthcare provider compassion, resilience, and patient care. Journal of Compassionate Health Care, 1(1) doi:10.1186/s40639-014-0005-9

Shahar, B., Szsepsenwol, O., Zilcha-mano, S., Haim, N., Zamir, O., Levi-Yeshuvi, S., & Levit-Binnun, N. (2015). A wait-list randomized controlled trial of loving-kindness meditation programme for self-criticism. Clinical Psychology & Psychotherapy, 22(4), 346-356. doi:10.1002/cpp.1893

Shaltout, H. A., Tooze, J. A., Rosenberger, E., & Kemper, K. J. (2012). Time, touch, and compassion: Effects on autonomic nervous system and well-being. Explore-the Journal of Science and Healing, 8(3), 177-184. doi:10.1016/j.explore.2012.02.001

SHAPIRO, D. (1992). A preliminary-study of long-term meditators - goals, effects, religious orientation, cognitions. Journal of Transpersonal Psychology, 24(1), 23-39.

Skipper, T., O'Donovan, A., Conlon, E., & Clough, B. (2015). An examination of the factor structure of the effects of meditation scale. Personality and Individual Differences, 86, 57-62. doi:10.1016/j.paid.2015.06.007

Stell, A. J., & Farsides, T. (2015). Brief loving-kindness meditation reduces racial bias, mediated by positive other-regarding emotions. Motivation and Emotion,

Tonelli, M. E., & Wachholtz, A. B. (2014). Meditation-based treatment yielding immediate relief for meditation-naive migraineurs. Pain Management Nursing, 15(1), 36-40. doi:10.1016/j.pmn.2012.04.002

Van Gordon, W., Shonin, E., Sumich, A., Sundin, E. C., & Griffiths, M. D. (2014). Meditation awareness training (MAT) for psychological well-being in a sub-clinical sample of university students: A controlled pilot study.Mindfulness, 5(4), 381-391. doi:10.1007/s12671-012-0191-5

Wallmark, E., Safarzadeh, K., Daukantaite, D., & Maddux, R. E. (2013). Promoting altruism through meditation: An 8-week randomized controlled pilot study. Mindfulness, 4(3), 223-234. doi:10.1007/s12671-012-0115-4

Weng, H. Y., Fox, A. S., Hessenthaler, H. C., Stodola, D. E., & Davidson, R. J. (2015). The role of compassion in altruistic helping and punishment behavior. PLoS ONE [Electronic Resource], 10(12), e0143794. doi:http://dx.doi.org/10.1371/journal.pone.0143794

Weng, H. Y., Fox, A. S., Shackman, A. J., Stodola, D. E., Caldwell, J. Z. K., Olson, M. C., . . . Davidson, R. J. (2013). Compassion training alters altruism and neural responses to suffering. Psychological Science, 24(7), 1171-1180. doi:10.1177/0956797612469537

Weytens, F., Luminet, O., Verhofstadt, L. L., & Mikolajczak, M. (2014). An integrative theory-driven positive emotion regulation intervention. Plos One, 9(4), e95677. doi:10.1371/journal.pone.0095677

Wheeler, E. A. & Lenick, N. W. (2014). Brief compassion meditation and recall of positive-emotion words. Journal of Articles in Support of the Null Hypothesis, 11(2), 12.

Williams AL, Selwyn PA, Liberti L, Molde S, Njike VY, McCorkle R, . . . Katz DL. (2005). A randomized controlled trial of meditation and massage effects on quality of life in people with late-stage disease: A pilot study. Journal of Palliative Medicine, 8(5), 939-952.

Woods, H., & Proeve, M. (2014). Relationships of mindfulness, self-compassion, and meditation experience with shame-proneness. Journal of Cognitive Psychotherapy, 28(1), 20-33.

Xu, J., Vik, A., Groote, I. R., Lagopoulos, J., Holen, A., Ellingsen, O., . . . Davanger, S. (2014). Nondirective meditation activates default mode network and areas associated with memory retrieval and emotional processing.Frontiers in Human Neuroscience, 8, 86. doi:10.3389/fnhum.2014.00086
